# Four New Iridoid Metabolites Have Been Isolated from the Stems of *Neonauclea reticulata* (Havil.) Merr. with Anti-Inflammatory Activities on LPS-Induced RAW264.7 Cells

**DOI:** 10.3390/molecules24234271

**Published:** 2019-11-23

**Authors:** Fang-Pin Chang, Shyh-Shyun Huang, Tzong-Huei Lee, Chi-I Chang, Tzong-Fu Kuo, Guan-Jhong Huang, Yueh-Hsiung Kuo

**Affiliations:** 1 The Ph.D Program for Cancer Biology and Drug Discovery, China Medical University and Academia Sinica, Taichung 404, Taiwan; u101049002@cmu.edu.tw; 2 School of Pharmacy, China Medical University, Taichung 404, Taiwan; sshuang@mail.cmu.edu.tw; 3 Institute of Fisheries Science, National Taiwan University, Taipei 106, Taiwan; thlee1@ntu.edu.tw; 4 Department of Biological Science and Technology, National Pingtung University of Science and Technology, Pingtung 912, Taiwan; changchii@mail.npust.edu.tw; 5 Department of Post-Baccalaureate Veterinary Medicine, Asia University, Taichung 413, Taiwan; tzongfu@asia.edu.tw; 6 Department of Chinese Pharmaceutical Sciences and Chinese Medicine Resources, China Medical University, Taichung 404, Taiwan; 7 Department of Biotechnology, Asia University, Taichung 413, Taiwan; 8 Chinese Medicine Research Center, China Medical University, Taichung 404, Taiwan

**Keywords:** *Neonauclea reticulata*, iridoid, bis-iridoid, neonanin, reticunin, RAW264.7

## Abstract

One new iridoid, namely neonanin C (**1**) one monocyclic iridoid ring-opened derivative namely neonanin D **(2)**, two new bis-iridoid derivatives namely reticunin A (**3**) and reticunin B (**4**) with sixteen known compounds (**5**–**20**) were isolated from the stems of *Neonauclea reticulata* (Havil.) Merr. These new structures were determined by the detailed analysis of spectroscopic data and comparison with the data of known analogues. Compounds **1**–**20** were evaluated for inhibition of nitric oxide (NO) production in lipopolysaccharide (LPS)-stimulated RAW264.7 macrophages cell line. The results showed that all compounds exhibited no obvious cytotoxicity compared to the control group and five compounds including isoboonein (**7**), syringaresinol (**10**), (+)-medioresinol (**12**), protocatechuic acid (**14**) and *trans*-caffeic acid (**15**) exhibited inhibitory activities with IC_50_ values at 86.27 ± 3.45; 9.18 ± 1.90; 76.18 ± 2.42; 72.91 ± 4.97 and 95.16 ± 1.20 µg/mL, respectively.

## 1. Introduction

The genus *Neonauclea* (Rubiaceae) includes 69 species of diverse trees and shrubs [1]. The plants of this genus are mainly distributed in the Malesian region, extending from India to Vietnam and from Southeast Asia to the northern limits of Australia [2]. *Neonauclea reticulata* (Havil.) Merr. *(N. reticulata)* is a large evergreen tree and the only species found in Taiwan, which is located in the forests at low elevations of southern Taiwan, such as Kaohsiung, Pingtung or Orchid [3]. *N. reticulata* is an important folk plant for Yami people (Tao) who used this plant as a resource to build the keels of bows and sterns in planked boats when they celebrated the flying fish festival on Orchid Island [4]. Phytochemical studies of the genus *Neonauclea* have been rare and anthraquinones [5], alkaloids [6], triterpenes [7] and saponins [8] have been isolated from previous chemical investigations. In our previous study, which is the only study that has mentioned phytochemical investigations about this plant, iridoids, lignans and phenolics were isolated from *N. reticulata* [9]. As for pharmacological activity, two studies have shown *N. reticulata* can be cytotoxic to Hep cells [9], used to guard against ultraviolet B (UVB) and protect human skin fibroblast cells [10]. Further investigations of the chemical and pharmacological properties of *N. reticulata* are urgently needed.

Inflammation is a complex immune reaction that is triggered by the body during microbial invasion, physical damage or activated by some immune cells such as macrophages. These trigger factors may induce redness, fever, pain and other symptoms in the human body [11]. Sometimes, an appropriate inflammation reaction is beneficial to activate the automatic defense response in the human body for healing and recovery. Unfortunately, excessive defense responses will lead to other human diseases, including Alzheimer’s disease, cardiovascular diseases, type 2 diabetes [12], cancers and organ dysfunction [13]. Lipopolysaccharide (LPS) is the main component of the outer membrane of Gram-negative bacteria and is also known as an endotoxin, which rapidly activates macrophages and stimulates the secretion of pro-inflammatory cytokines as well as inflammatory mediators such as nitric oxide (NO) and prostaglandin E2 (PGE2). Because NO plays an important role in the process of inflammation progress, the ability to inhibit production of NO could be a potential indicator for anti-inflammatory [14].

The aim of this study is to investigate anti-inflammatory ability of *N. reticulata* by using *in-vitro* NO inhibitory assay in the LPS-stimulated RAW264.7 macrophage cell line and to identify the compounds that mainly contribute to the anti-inflammatory ability and expectantly it can provide the preliminary information for future research in drug discovery of anti-inflammatory agent.

## 2. Results and Discussions

### 2.1. Isolation and Structural Elucidation

**Figure 1:**
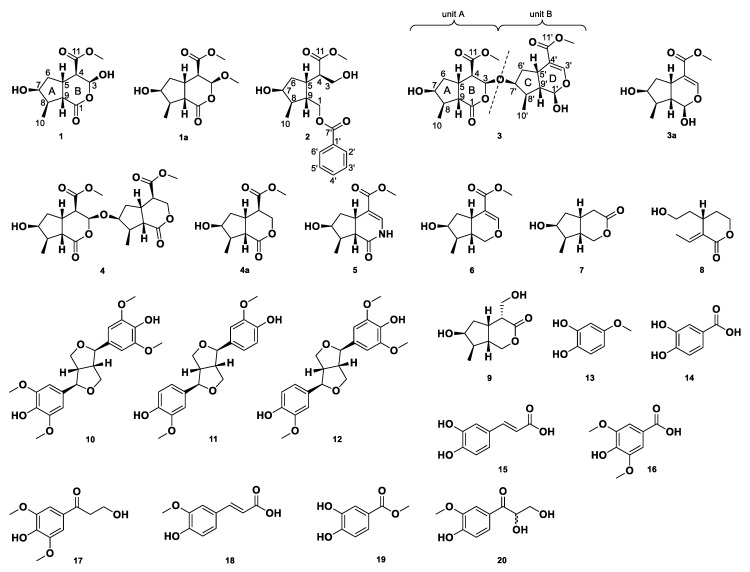
The chemical structures of compound **1**–**20** from *Neonauclea reticulata* (Havil.) Merr.

In this study, we tested four fractions (MeOH extract, EtOAc fraction, BuOH fraction and H_2_O fraction that have been described in our previous study) for their cytotoxic activity and inhibition ability of NO produce against the RAW264.7 cells stimulated by LPS. The results showed that four fractions had no significant cytotoxicity. The MeOH extract and EtOAc fraction exhibited dose-dependent inhibition abilities. (Half maximal inhibitory concentration; IC_50_ = 465.71 ± 6.94 μg/mL & 350.74 ± 8.64 μg/mL). In contrast, BuOH and H_2_O fractions showed no inhibition of NO. Twenty compounds were isolated from the EtOAc fraction by silica gel column chromatography and normal-phase, semi-preparative, high-performance liquid chromatography (HPLC). Among these compounds, we isolated one new iridoid, which namely neonanin C (**1**) (neonanin A and B have been reported in our previous study [9]), one monocyclic iridoid ring-opened derivative namely neonanin D (**2**), two new bis-iridoid derivatives namely reticunin A (**3**), reticunin B (**4**) and sixteen known compounds, including strychnovoline (**5**) [15], siphonostegiol (**6**) [16], isoboonein (**7**) [17], floribundane B (**8**) [18], alyxialactone (**9**) [19], syringaresinol (**10**) [20], (+)-pinoresinol (**11**), (+)-medioresinol (**12**) [21], 4-methoxycatechol (**13**) [22], protocatechuic acid (**14**) [23], *trans*-caffeic acid (**15**) [24], syringic acid (**16**) [25], 3-hydroxy-1-(4-hydroxy 3,5-dimethoxyphenyl)-1-propanone (**17**) [26], ferulic acid (**18**) [27], protocatechuic acid methyl ester (**19**) [28], C-veratroylglycol (**20**) [29]. The structures of the new compounds were determined through spectral analyses, including IR, UV, 1D- and two-dimensional (2D)-NMR, as well as HR-ESI-MS data. The known compounds identified were compared with the published NMR spectral literatures. All structures are shown in Figure 1. In this study, we described the detailed structures of the new compounds and the activities of compounds **1**–**20**.

**Figure 2:**
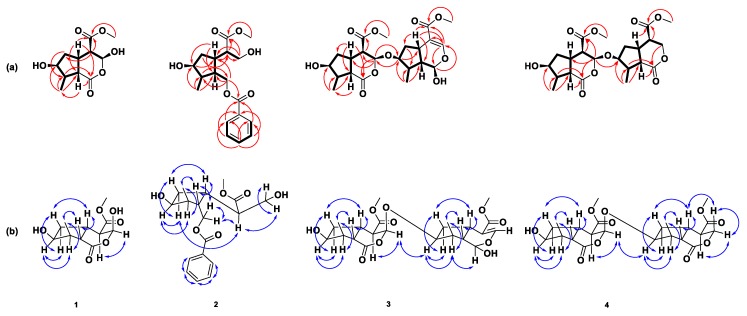
(**a**) Significant COSY (bold line) correlations and (HMBC) (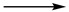) correlations for compounds **1**–**4**; (**b**). Significant NOESY (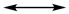) correlations of compounds **1**–**4**.

Compound **1** was obtained as a colorless oil. ${\left[\alpha \right]}_{D}^{28}$ + 22 (*c* = 0.17, MeOH). The molecular weight was determined by HR-ESI-MS, which showed an [M − H]^+^ ion at an *m*/*z* of 243.0870 (calculated for C_11_H_15_O_6_ 243.0863), indicating four degrees of unsaturation. The IR spectrum displayed the presence of hydroxyl (3365 cm^−1^) and γ-lactone and ester (1730 cm^−1^) functionalities. The ^1^H spectra of compound **1** (Table 1) presented a doublet methyl group δ_H_ 1.28 (d, *J* = 7.0, H-10) and a singlet carbonylmethoxy group at δ_H_ 3.78 (s, OMe); one pair of geminal coupling methylene groups at δ_H_ 1.45 (td, *J* = 10.8, 3.4, H-6α) and δ_H_ 2.33 (m, H-6β); six methine protons including four aliphatic methines at δ_H_ 2.59 (dd, *J* = 10.9, 2.1, H-4), δ_H_ 2.88 (t, *J* = 10.9, H-9), δ_H_ 2.30 (m, H-8) and δ_H_ 3.32 (m, H-5); one oxymethine at δ_H_ 4.14 (q, *J* = 3.4, H-7) and one hemiacetal methine at δ_H_ 5.90 (brs, H-3). The ^13^C-NMR spectrum (Table 1) showed 11 signals with the help of distortionless enhancement by polarization transfer (DEPT) and heteronuclear single quantum coherence (HSQC) experiment in the presence of two carbonyl groups δ_C_ 173.7 (C-1) and δ_C_ 170.6 (C-11). The correlation spectroscopy (COSY) (Figure 2a) correlations with H-4 (δ_H_ 2.59)/H-3 (δ_H_ 5.90) and H-5 (δ_H_ 3.32); H-5 (δ_H_ 3.32)/H-6 (δ_H_ 1.45 and δ_H_ 2.33) and H-9 (δ_H_ 2.88); H-6 (δ_H_ 1.45 and δ_H_ 2.33)/H-7 (δ_H_ 4.14); H-7 (δ_H_ 4.14)/H-8 (δ_H_ 2.30); H-8 (δ_H_ 2.30)/H-9 (δ_H_ 2.88) and H-10 (δ_H_ 1.28), these findings confirms that a cyclopentanyl moiety with a methyl group and a hydroxyl group in the structure. The key of heteronuclear multiple bond coherence (HMBC) correlations from H-4 (δ_H_ 2.59)/C-6 (δ_C_ 42.0) and C-11 (δ_C_ 170.6); H-5 (δ_C_ 3.32)/C-9 (δ_C_ 46.2), C-11 (δ_C_ 170.6) and C-1 (δ_C_ 173.7); H-9 (δ_C_ 2.88)/C-1 (δ_C_ 173.7), C-6 (δ_C_ 42.0) and C-10 (δ_C_ 14.1), From the information of ^1^H-NMR, DEPT and HSQC spectrums, we determined that C-3 (δC 94.7) is a carbon of hemiacetal methine and the chemical shift was lower than the general range, which proves that the C-3 carbon is connected to the two oxygenated groups. Further, these correlations combined with the IR spectrum indicated that there was a γ-lactone and ester carbonyl group connected with a pyranyl moiety in the structure. Apart from this, the correlation between OMe (δ_H_ 3.78) to C-11 (δc 170.6) supported that a methoxy group was located at C-11. Summarizing the currently available information, we are getting more certain that compound **1** is a cyclopenta [*c*] pyran-type iridoid structure. Thus, the ^1^H and ^13^C-NMR data of 1 were very similar to neonanin B (compound **1a**) [9], except there was only one methoxy group in compound **1**. The functional group in the C-3 position of neonanin B was a methoxy group, while a hydroxyl group was located in the C-3 position in compound **1**. The proton of H-4 exhibited two coupling constants, with *J* = 10.9, 2.1 but the coupling constant of H-3 was not directly shown and was combined with the nuclear overhauser effect spectroscopy (NOESY) correlations (Figure 2b). Even so, we can be sure that the coupling constants and correlations of the NOESY spectrum were also very similar to neonanin B by comparing them to the literature. Thus, the evidence concluded that both of H-3 and H-4 were located in the same phase; the difference is H-3 was located in α-pseudoequatorial and H-4 in α-pseudoaxial configuration. The coupling constant with *J* = 10.9 between H-4 and H-5 and suggested that H-5 is in a β-pseudoaxial orientation [30]. This also confirmed H-6β, H-9 and methyl group in the β-configuration, while H-6α, H-7 and H-8 were α-oriented. By consolidating the above-mentioned results and comparing them to the literature, to the best of our knowledge, compound **1** has been isolated for the first time and assigned to be neonanin C.

**Figure 3:**
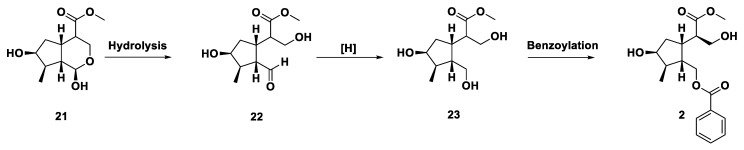
Proposed biosynthetic sequence of neonanin D (**2**).

Compound **2** was obtained as a colorless oil. ${\left[\alpha \right]}_{D}^{29}$ + 16 (*c* = 0.10, MeOH). The molecular weight was determined by HR-ESI-MS, which showed an [M + Na]^+^ ion at an *m*/*z* of 359.1457 (calculated for C_18_H_24_O_6_Na 359.1465), indicating seven degrees of unsaturation. The IR spectrum displayed the presence of hydroxyl (3387 cm^−1^), an ester carbonyl and conjugated ester carbonyl groups (1720 cm^−1^). The UV spectrum showed two *λ*max absorption at 228 nm (logε 3.9) and 273 nm (logε 3.4). The ^1^H and ^13^C NMR data (Table 1) of this compound showed three moieties. The first moiety exhibited a hydroxyl methyl group at δ_H_ 3.89 (dd, *J* = 11.0, 3.6, H-3), δ_H_ 3.77 (dd, *J* = 11.0, 6.8, H-3), δ_C_ 63.6 (C-3), a carbonylmethoxy group at δ_H_ 3.67 (s, 11-OMe), δ_C_ 52.2 (11-OMe), δ_C_ 175.4 (C-11) together with a methine proton at δ_H_ 2.70 (ddd, *J* = 10.7, 6.8, 3.6, H-4), δ_C_ 48.3 (C-4), these NMR signals evidenced that a methyl 3-hydroxypropanoate group with a substituent was located at the C-2 position. The second moiety exhibited NMR signals at δ_H_ 8.00 (d, *J* = 7.5, H-2′ & H-6′), δ_C_ 129.7 (C-2′ & C-6′); δ_H_ 7.57 (t, *J* = 7.5, H-4′), δ_C_ 133.3 (C-4′) and δ_H_ 7.45 (t, *J* = 7.5, H-3′ & H-5′), δ_C_ 128.7 (C-3′ & C-5′), δ_C_ 130.4 (C-1′) and δ_C_ 166.8 (C-7′) combined with methylene ester at δ_H_ 4.44 (dd, *J* = 11.7, 4.9, H-1), δ_H_ 4.21 (dd, *J* = 11.7, 5.7, H-1), δ_C_ 65.4 (C-1). Then, UV and IR absorption data confirmed the second moiety being a methylenyl benzoate group. By compassion of 1D and 2D NMR spectra in Figure 2a and Table 1, the third moiety A ring of compound **2** was similar to the A ring of compound **1**, being a cyclopentane iridoid moiety. Analysis of the COSY experiment (Figure 2a) permitted the establishment that H-4 (δ_H_ 2.70) in methyl 3-hydroxypropanoate moiety was correlated with H-5 (δ_H_ 2.99) and HMBC correlation with C-5 (δ_C_ 37.0); H-6α (δ_H_ 1.65) was correlated with C-4 (δ_C_ 48.3). The result proved that methyl 3-hydroxypropanoate with its C-2 position was located at C-5 of the A ring. Meanwhile, the H-1 of the methylenyl benzoate group was coupled with H-9 in the A ring and also exhibited correlations with C-5 (δ_C_ 37.0), C-8 (δ_C_ 42.4) and C-9 (δ_C_ 44.9) in HMBC spectra. This evidence gave the result that methyl 3-hydroxypropanoate was positioned at C-9 of the A ring. The most stable conformer of compound **2** is shown in Figure 2b. There are two important points that can be determined from the NMR spectra: one in H-4 showing NOESY correlation with H-6α and second proved the coupling constant between H-4 and H-5 with *J* = 10.7, which showed a larger coupling constant, except that it was anti-coplanar. The proposed biosynthetic pathway is illustrated by positions 1 and 2 of dihydrologanin (**21**) being hydrolyzed to compound **22**, **22** being reduced to compound **23** and finally, compound **23** being benzylated to compound **2** as shown in Figure 3. Accordingly, compound **2** was established as depicted and given the name neonanin D.

Compound **3** was obtained as a colorless oil. ${\left[\alpha \right]}_{D}^{28}$ + 32 (c = 0.07, MeOH). The molecular weight was determined by HR-ESI-MS, which showed an [M − H]^+^ ion at *m*/*z* of 453.17553 (calculated for C_22_H_29_O_10_ 453.17752), indicating eight degrees of unsaturation. The IR spectrum displayed the presence of hydroxyl (3387 cm^−h^) and ester lactone and conjugated ester carbonyl groups (1722 cm^−1^). The UV spectrum showed the *λ*_max_ absorption at 236 nm (logε 3.6). The ^1^H and ^13^C spectra of this compound (Table 2) exhibited two iridoid moieties: units A and B. The proton and carbon signals of unit A showed similar chemical shifts compared to compound **1** by ^1^H-NMR, ^13^C-NMR and HSQC spectrums, which indicated that structure of unit A was neonanin C. Among them, the chemical shift at position 3 in the compound **3** was different compared to compound **1**. The proton H-3 upshifted to δ_H_ 5.63 (d, *J* = 2.6); carbon signal downshifted to δ_C_ 98.3 (C-3), this suggests that an alkoxy group is connected in position 3 instead of a hydroxyl group. For unit B, the ^1^H-NMR spectrum indicated the presence of an olefinic proton at δ_H_ 7.40 (d, *J* = 2.1, H-3′), an hemiacetal proton at δ_H_ 4.93 (d, *J* = 5.6, H-1′), one oxymethine at δ_H_ 4.20 (m, H-7′), a carbonylmethoxy group at δ_H_ 3.71 (s, 11′-OMe) and a doublet methyl group at δ_H_ 1.08 (d, *J* = 6.9, H-10′). Moreover, the ^13^C-NMR spectrum in combination with IR and UV spectra suggested the existence of an α, β-unsaturated ester carbon at δ_C_ 167.6 (C-11′), the signals at δ_C_ 151.5 (C-3′), δ_C_ 111.8 (C-4′) for double bonds and β-hydroxy pyranose anomeric carbon at δ_C_ 95.7 (C-1′), one methoxy carbon at δ_C_ 51.4 (11′-OMe) and methyl at δ_C_ 13.9 (C-10′) can be observable in this moiety. Along with the main protons correlated in the COSY spectrum (Figure 2a) from ring C, the existence of a cyclopentane skeleton is suggested. The main HMBC correlations (Figure 2a) of the carbonylmethoxy group were represented by the α, β-unsaturated bond from ring D by 11′-OMe (δ_H_ 3.71) to C-11′ (δ_C_ 167.6) and H-3′ (δ_H_ 7.40) to C-1′(δ_C_ 95.7), C-4′ (δ_C_ 111.8), C-11′ (δ_C_ 167.6). Ring D linked with ring C in unit B was confirmed as such H-1′ (δ_H_ 4.93) to C-3′ (δ_C_ 151.5), C-5′ (δ_C_ 31.7); H-5′ (δ_H_ 2.93) to C-3′ (δ_C_ 151.5), C-4′ (δ_C_ 111.8). This unit B is almost identical to loganetin (Compound **3a**), which was isolated in our previous study. The HMBC showed that these two units were linked by an O-linkage, as it showed correlations between H-3 (δ_H_ 5.63) in unit A to C-7′ (δ_C_ 80.4) in unit B and H-7′ (δ_H_ 4.20) to C-3 (δ_C_ 98.3), therefore, this suggests that compound **3** is a bis-iridoid with a C-3-O-C-7′ ether linkage-type structure. In NOESY correlation results, unit A suggested a stereochemistry similar to neonanin C. Protons from ring C also showed the same NOESY results as those of ring A. In the literature review, the reports present the α-configuration at proton H-1′ of the loganetin moiety [31]. NOESY cross peak between H-3 (δ_H_ 5.63) to H-7′ (δ_H_ 4.20) confirmed that these two protons were located in α form and gave this compound the most stable conformation, as shown in Figure 2b. This correlation supported the above stereostructure configuration. Accordingly, the structure of compound **3** was named reticunin A.

Compound **4** was obtained as colorless oil. ${\left[\alpha \right]}_{D}^{28}$ + 27 (*c* = 0.05, MeOH). The molecular weight was established by HR-ESI-MS, which showed an [M − H]^+^ ion at *m*/*z* of 453.1767 (calculated for C_22_H_29_O_10_ 453.1755), indicating eight degrees of unsaturation. The IR spectrum displayed the presence of hydroxyl (3414 cm^−1^) and γ-lactone (1734 cm^−1^) groups. Comparison of ^1^H and ^13^C-NMR values and analysis of the 2D spectrum showed that **3** and **4** contained the same skeleton and unit A moiety; the difference between **3** and **4** should be the iridoid derivative moiety located at unit B. On the main ^1^H and ^13^C-NMR spectrum (Table 2) of unit B, one CH_2_OR group at δ_H_ 4.25 (dd, *J* = 11.3, 9.8, H-3′β) and δ_H_ 4.45 (dd, *J* = 11.3, 3.3, H-3′α); δ_C_ 67.5 (C-3′), one oxymethine at δ_H_ 4.23 (m, H-7′); δ_C_ 81.0 (C-7′), one carbonylmethoxy group at δ_H_ 3.74 (s, 11′-OMe); δ_C_ 52.6 (11′-OMe) and one doublet methyl group at δ_H_ 1.19 (d, *J*6.8, H-10′); δ_C_ 14.7 (C-10′) were shown. Besides, secondary carbon δ_C_ 37.3 (C-6′) and tertiary carbon δ_C_ 37.4 (C-5′) could surely identified by DEPT. The cyclopentanyl moiety of unit B was also assigned by using COSY experiment (Figure 2a). The connectivity between A and B units was found to be an *O*-linkage between position 3 to position 7′ by δ_H_ 5.59 (H-3) of unit A correlated to carbon δ_C_ 81.0 (C-7′) in unit B from HMBC spectrum (Figure 2a). The position of two carbonyl carbon δ_C_ 171.2 (C-11′) and δ_C_ 173.3 (C-1′) were also deduced by the HMBC correlation form δ_H_ 4.45 (H-3′α), δ_H_ 4.25 (H-3′β), δ_H_ 2.64 (H-9′) and δ_H_ 2.40 (H-8′) to δ_C_ 173.3 (C-1′); δ_H_ 2.84 (H-5′), δ_H_ 2.52 (H-4′) and δ_H_ 3.74 (11′-OMe) to δ_C_ 171.2 (C-11′) in ring D. Thus, the moiety of unit B could be compared to literature and assigned as 6-hydroxy-7-methyl-1-oxo-4-carbomethoxyoctahydrocyclopenta[*c*]pyran (compound **4a**) [32]. The relative configuration of unit A of **4** was confirmed with **3** and got the same correlation results. The NOESY spectrum of ring C also exhibited a similar cross peak signal to **3** and the coupling constants from ring D, *J*_3′β, 4′_ = 9.8 and *J*_3′α, 4′_ = 3.3 suggested that the carbonylmethoxy group and H-3′β were in β-form, while H-4′ and H-3′α were in α-form. Finally, H-3 (δ_H_ 5.59) was correlated to H-7′ (δ_H_ 4.23) located in α form are the most stable conformation for compound **4**. Thus, the structure of compound **4** was concluded to be reticunin B.

### 2.2. In-Vitro Cell Viability and NO Inhibition Activity of the Compounds ***1**–**20***

In this study, nine iridoids (**1**–**9**), three lignans (**10**–**12**) and eight aromatic rings (**13**–**20**) were exposed to different concentrations (6.25, 12.5, 25, 50 and 100 μg/mL) for testing their cell viability, while the control group was only stimulated by LPS. There were no obvious effects of all compounds on cell viability determined by MTT assay. Furthermore, twenty compounds were tested for the inhibitory effects on NO production by LPS-stimulated RAW 264.7 cells. As shown in Table 3, Isoboonein (**7**), (+)-medioresinol (**12**), protocatechuic acid (**14**) and *trans*-caffeic acid (**15**) found to have IC_50_ values compared with positive drug (indomethacin) at 86.27 ± 3.45 (*p* < 0.001); 76.18 ± 2.42 (*p* < 0.001); 72.91 ± 4.97 (*p* < 0.005) and 95.16 ± 1.20 µg/mL (*p* < 0.001), respectively. Syringaresinol (**10**) exhibited better IC_50_ values at 9.18 ± 1.90 µg/mL (*p* < 0.001) compared with indomethacin. The cell viability (%) and nitric oxide production (%) in each concentration of these five compounds were presented in Figure S32. These compounds may possibly contribute to the NO inhibition activity of *N. reticulata*. Indomethacin was used as a positive control in this study, which is a clinically effective anti-inflammatory medication that inhibits COX-1 and COX-2 and reduces prostaglandin synthesis [33]. The IC_50_ value of indomethacin was 46.71 ± 3.14 µg/mL. From literature investigation, NO which was produced by nitric oxide synthase (iNOS) was involved in the pro-inflammatory responses [34], besides, COX-2, TNF-α and IL-1β, these pro-inflammatory mediators also were regulated by activation of the transcription factor NF-κB [35]. The results of LPS stimulated RAW264.7 cells would cause the phosphorylation of p38 MAPK and JNK1/2, leading NF-κB activation demonstrated by Elke Cario in 2000 [36]. Also, oxidative stress was the reason to activate a variety of mediators in inflammatory progress [37]. Compound **10** could interference with JNK and p38 phosphorylation to down-regulating NF-κB expression and reducing levels of iNOS, COX-2 and TNF-α [38] and demonstrated as anti-oxidative by DPPH and hydroxyl radical scavenging assay [39]. Compound **14** may inhibit the expression of TNF-α, IL-β and COX-2 by regulating NF-κB [35], compound **15** could mediated NO production by down-regulating NF-κB, p38 MAP kinase and JNK1/2 [40]. These two compounds could be anti-oxidative materials by confirmed with DPPH and ABTS radical scavenging assay [41]. The detailed mechanism of (+)-medioresinol (**12**) is still unclear but there has some reference indicated that this compound was found to have antioxidant [42] and inhibited NO production in LPS-stimulated RAW cells [43]. Herein, inhibition of these mediators production or scavenging oxidative stress abilities will be the main reason for compounds to have anti-inflammatory properties. In our knowledge, isoboonein (**7**) has been shown to have antibacterial and antitumor activity in previous studies [44], while anti-inflammatory activity is still unknown. Therefore, this is the first study to investigate the preliminary anti-inflammatory activity of isoboonein by testing NO inhibition ability. Our work only presented very preliminary cell viability and NO inhibition activity. The anti-inflammatory mechanisms of compound **10**, **14** and **15** have been careful described in the literatures. In future works, the experiments to test NF-kB, TNF-α, IL-1β, IL-6, MAPK and PGE2 regulated abilities of compound **7** and **12** and also antioxidant ability of compound **7** are needed. These assays can increase the two compounds’ credibility of anti-inflammatory and also explore the mechanisms.

## 3. Materials and Methods

### 3.1. General

The optical rotation data were measured in MeOH with a Jasco P-2000 Polarimeter (JASCO Inc., Tokyo, Japan). The infrared spectra were acquired on Shimadzu IR Affinity-1S Fourier Transform Infrared Spectrophotometer. The UV spectra were obtained from LAMBDA 265 UV/Vis Spectrophotometer (PerkinElmer Inc., Waltham, MA, USA). The 1D and 2D-NMR spectra were recorded with a Bruker Avance 500 FT-NMR spectrometer (Bruker Inc., Bremen, Germany). The HR-ESI-MS data were generated at the Mass Spectrometry Laboratory of the Chung Hsing University with a Thermo LTQ Orbitrap XL™ Hybrid Ion Trap-Orbitrap Mass Spectrometer (Thermo Scientific Inc., Waltham, MA, USA). Column chromatography was performed using LiChroCART Si 5 µM gel (Merck, Darmstadt, Germany) and Sephadex LH-20 (GE Healthcare Life Sciences Inc., Marlborough, MA, USA). The TLC (thin-layer chromatography) analysis was carried out using aluminum pre-coated Si plates (Silica Gel 60 F-254; Merck). The spots were visualized using a UV lamp at λ = 254 nm and detected by spraying with 10% H_2_SO_4_ alcohol solution and heating at 125 °C. Semi-preparative HPLC was performed using a normal phase column (Luna 5μm Silica 100 Å, 250 × 10 mm; Phenomenex Inc.) on a Precision Instruments IOTA 2 Refractive Index Detector system.

### 3.2. Chemicals

The solvents used to open the column isolation (Silica gel and Sephadex LH 20 gel column) in the study, such as *n*-hexane, dichloromethane, chloroform, ethyl acetate, acetone and methanol, were of ACS grade. The *n*-hexane, dichloromethane, chloroform, ethyl acetate and acetone used for HPLC isolation, which was of HPLC grade and the deuterated solvents for NMR measurement (CDCl_3_ and CD_3_COCD_3_) were purchased from the branch of Merck in Taipei, MTT (3-(4,5-dimethylthiazol-2-yl)-2,5-diphenyltetrazolium bromide), LPS (*Escherichia coli* 055:B5), indomethacin and other chemicals were purchased from Sigma Chemical Co. (St. Louis, MO, USA). Minimum essential media (MEM), trypsin–EDTA, fetal bovine serum (FBS), penicillin/streptomycin, non–essential amino acids (NEAA) and sodium pyruvate were obtained from Gibco (BRL life Technologies, Grand Island, NY, USA).

### 3.3. Plant Material

The stems of *Neonauclea reticulata* were collected from Nan Ren Mountain, Pingtung, Taiwan, in August 2012 and identified by Yau Lun Kuo (Professor, Department of Forestry, National Pingtung University of Science and Technology, Pingtung, Taiwan). A voucher specimen (CMU-NR-201208) was deposited at the School of Chinese Pharmaceutical Sciences and Chinese Medicine Resources.

### 3.4. Extraction and Isolation

The air-dried stems of *Neonauclea reticulata* (9.0 kg) were extracted with MeOH (50 L each for seven days) three times at room temperature. The extracts were filtered; the filtrate was evaporated under reduced pressure at 35 °C with a rotavapor to obtain the MeOH extracts (365 g). The MeOH extracts were suspended in distilled water and successively solvent partitioned with ethyl acetate and *n*-buthanol (1:1,v/v), yielding soluble fraction of ethyl acetate (EtOAc) (100 g), *n*-buthanol (BuOH) (152 g) and water (H_2_O) (98 g). The EtOAc solution fraction was to passage over by column chromatography (CC) (2.0 kg of SiO_2_, 70–230 mesh; n-hexane/EtOAc/methanol gradient) to allow 32 fractions, Fr.1–Fr.32.

Fr.22 (4.6 g) was re-separated by sephadex LH-20 (250 g; CHCl_3_/MeOH = 3/7) to produce 11 fractions. Fr.22-6 (1.47 g) was re-separated by silica gel column chromatography (30 g of SiO_2_, 70–230 mesh; CHCl_3_/EtOAc (25%)) and to obtain 21 subfractions, Fr.22-6-10 (56.7 mg) purified by normal-phase HPLC (*n*-hexane/acetone (40%)) to afford pure compound **7** (14.6 mg, *t*_R_ = 26 min); Fr.22-6-13 (725.69 mg) was further purified was further purified through a silica gel column (2 × 16.5 cm) to obtain 8 subfractions. Fr.22-6-13-3 (17.6 mg) purified by HPLC (*n*-hexane/acetone (40%)) to afford pure compound **6** (1.1 mg, *t*_R_ = 13 min) and **1** (1.4 mg, *t*_R_ = 24 min). Fr.22-7 (275 mg) was purified by HPLC (CHCl_3_/EtOAc (40%)) to obtain 13 subfractions. Fr.22-7-3 (15.13 mg) was purified by HPLC (*n*-hexane/acetone (40%)) to afford pure compounds **11** (1.5 mg, *t*_R_ = 13 min), **13** (2.5 mg, *t*_R_ = 17 min) and **5** (7.8 mg, *t*_R_ = 18 min). Fr.22-7 (275.0 mg) was re-separated by silica gel column chromatography (5.5 g of SiO_2_, 70–230 mesh; CHCl_3_/acetone (20%)) to afford 14 fractions. Fr.22-7-8 (7.4 mg) was purified by normal phase HPLC (*n*-hexane/acetone (30%)) to form pure compounds **10** (3.2 mg, *t*_R_ = 15 min) and **11** (2.0 mg, *t*_R_ = 26 min). Fr.22-9 (141.2 mg) was separated by normal phase HPLC (CHCl_3_/acetone (20%)) to afford 12 subfractions. Fr.22-9 (141.2 mg) was purified by normal phase HPLC (*n*-hexane/acetone (40%), resulting in pure compounds **19** (2.2 mg, *t*_R_ = 4 min), **18** (12 mg, *t*_R_ = 13 min), **16** (3 mg, *t*_R_ = 14 min) and **17** (2.5 mg, *t*_R_ = 16 min). Fr.22-11(11.5 mg) was purified by normal phase HPLC (CHCl_3_/acetone (22%) and afford compound **14** (2.3 mg, *t*_R_ = 16 min).

Fr.23 (1.2 g) was re-separated by sephadex LH-20 (140 g; CHCl_3_/MeOH = 3/7) to afford 6 fractions, Fr.23-3 (832.68 mg) was re-separated by silica gel column chromatography (16 g of SiO_2_, 70–230 mesh; CHCl_3_/EtOAc (25%)) to afford 13 subfractions. Fr.23-3-1 (34 mg) was purified by normal phase HPLC (*n*-hexane/acetone (40%), resulting in pure compounds **4** (0.8 mg, *t*_R_ = 6 min), **3** (2 mg, *t*_R_ = 7 min), **20** (1.9 mg, *t*_R_ = 35 min) and **10** (3.7 mg, *t*_R_ = 44 min). Fr.23-4 (48.5 mg) was re-separated by silica gel column chromatography (970 mg of SiO_2_, 70–230 mesh; CHCl_3_/acetone (45%)) to afford 2 subfractions. Fr.23-4-1 (37.8 mg) was purified by normal phase HPLC (CHCl_3_/acetone (45%) to get compounds **15** (2 mg, *t*_R_ = 21 min) and **2**(1.2 mg, *t*_R_ = 40 min).

Fr.25 (6.6 g) was re-separated by sephadex LH-20 (300 g; CHCl3/MeOH = 3/7) to produce 26 fractions. Fr.25-14 (934.5 mg) was re-separated by silica gel column chromatography (1.9 g of SiO_2_, 70–230 mesh; CH_2_Cl_2_/acetone (30%)) to get compound **9** (35.3 mg)

Neonanin C (**1**): colorless oil; ${\left[\alpha \right]}_{D}^{28}$ +22 (*c* = 0.17, MeOH); IR (KBr) *ν*_max_: 3365, 2937, 1730, 1454 and 1026 cm^−1^; HR-ESI-MS *m*/*z* 243.0870 [M − H]^+^ (calculated for C_11_H_15_O_6_ at 267.0863); ^1^H-NMR (500 MHz, in CDCl_3_) and ^13^C-NMR (125 MHz, in CDCl_3_) are shown on Table 1.

Neonanin D (**2**): colorless oil; ${\left[\alpha \right]}_{D}^{29}$ + 16 (*c* = 0.10, MeOH); IR (KBr) *ν*_max_: 3387, 2945, 1720, 1274, 1028 and 715 cm^−1^; UV(MeOH) *λ*_max_ (log ε): 228 (3.9), 273 (3.4) nm; HR-ESI-MS *m*/*z* 359.1457 [M + Na]^+^ (calculated for C_18_H_24_O_6_Na at 359.1465); ^1^H-NMR (500 MHz, in CDCl_3_) and ^13^C-NMR (125 MHz, in CDCl_3_) are shown on Table 1.

Reticunin A (**3**): colorless oil; ${\left[\alpha \right]}_{D}^{28}$ + 32 (*c* = 0.07, MeOH); IR (KBr) *ν*_max_: 3387, 2931, 1722, 1633, 1446, 1176 and 1006 cm^−1^; UV (MeOH) λ_max_ (log ε): 236 (3.6) nm; HR-ESI-MS *m*/*z* 453.17553 [M + Na]^+^ (calculated for C_22_H_29_O_10_ 453.17752); ^1^H-NMR (500 MHz, in CDCl_3_) and ^13^C-NMR (125 MHz, in CDCl_3_) are shown on Table 2.

Reticunin B (**4**): colorless oil; M; ${\left[\alpha \right]}_{D}^{28}$ + 27 (*c* = 0.05, MeOH); IR (KBr) ν_max_: 3414, 2953, 2918, 2850, 1734, 1462 and 1180 cm^−1^; HR-ESI-MS *m*/*z* 453.1767 [M − H]^+^ (calculated for C_22_H_29_O_10_ 453.1755); ^1^H-NMR (500 MHz, in CDCl_3_) and ^13^C-NMR (125 MHz, in CDCl_3_) are shown on Table 2.

### 3.5. Cell Culture

RAW264.7 the mouse macrophages cell line (Bioresource Collection and Research Center (BCRC) Number: 60001) was obtained from Food Industry Research and Development Institute (Hsinchu, Taiwan). All of the cell lines were cultured in DMEM containing 10% (v:v) fetal bovine serum (FBS), penicillin and streptomycin (100 U/mL), 4 mM L-glutamine (100 U/mL). The cells were cultured in a humidified incubator under 5% CO_2_ at 37 °C. The RAW264.7 cells (5 × 10^5^ cells per mL) were seeded into plates for 24 h before treatment.

### 3.6. Cell Viability Assay

The *in-vitro* cell viability activity of MeOH extracts, partition fractions and pure compounds were determined by the MTT assay. RAW264.7 cells (5 × 10^4^/well) were seeded in 96-well plates and incubated for 24 h. The cells were treated with MeOH extracts and contain partition fractions in various concentrations (0, 62.5, 125, 250, 500 and 1000 μg/mL), compounds and positive control (0, 6.25, 12.5, 25, 50 and 100 μg/mL) in the presence or absence of LPS (100 ng/mL) for 24 h. After 24 h, the medium was replaced with a medium containing 0.5 mg/mL MTT solution and incubated at 37 °C for 4 h. After the end of the MTT reaction, we used 0.04 N HCl /isopropanol solution to dissolve the formazan crystals. The absorbance was measured at 570 nm using a microplate reader (Molecular Devices, LLC.). The cell viability was calculated and compared with the control group.

### 3.7. NO Assay

The supernatant collected from cell culture and mixed with an equal volume of Griess reagent (1% sulphanilamide, 0.1% naphthylethylenediamine dihydrochloride and 5% phosphoric acid) and the absorbance value was measured at 540 nm using an ELISA plate reader.

### 3.8. Statistical Analysis

The IC_50_ values for NO production were presented as mean values ± SD (standard deviations) of at least three independent experiments. The statistical significance of the differences was evaluated using Student’s *t*-test using IBM SPSS v.20.0 (SPSS Inc., Chicago, IL, USA). The percentage of NO production and cell viability were compared using variance analysis with GraphPad Prism 7.0 (GraphPad Software Inc., San Diego, CA, USA).

## 4. Conclusions

In conclusion, four iridoid derivatives, together with sixteen known compounds were identified in this work. Compounds **7**, **10**, **12**, **14**, **15** exhibited inhibitory activities with IC_50_ values compare to indomethacin (positive control). Syringaresinol (**10**) displayed the most NO inhibitory ability with IC_50_ values at 9.18 ± 1.90 µg/mL which is 5 fold less then indomethacin (positive control) (46.71±3.14μg/mL) and shows a significant difference. This is the first study that has provided preliminary evidence on the inhibition ability of NO produced by LPS-stimulated RAW 264.7 cells from *Neonauclea reticulata* (Havil.) Merr. According to our bioassay result and literatures, we propose that the position and numbers of methoxyl group [45] on the benzene ring in the structure of lignan constituents and aromatic compounds with catechol moiety [46] and carboxylic acid side chain [47] were important for this activity. NO is an important mediator in the process of inflammation and anti-inflammatory agents plays a key role in multiple chronic disease treatment; we believe these compounds from *N.reticulata* could be potential lead compounds in drug discovery. Although we only provide very preliminary result of anti-inflammatory effect, our findings still provide important information for researchers in future work and further study to explore anti-inflammatory mechanism is needed.

## Figures and Tables

**Table 1:** ^1^H-NMR and ^1^^3^C-NMR spectroscopic data of compounds **1**–**2** (in CDCl_3_, 500 MHz) ^a^.

Compounds	1		2	
Position	^1^H ^a^	^13^C	^1^H ^a^	^13^C
1		173.7	4.21 (dd, *J* = 11.7, 5.7)	65.4
			4.44 (dd, *J* = 11.7, 4.9)	
2				
3	5.90 (brs)	94.7	3.77 (dd, *J* = 11.0, 6.8)	63.6
			3.89 (dd, *J* = 11.0, 3.6)	
4	2.59 (dd, *J* = 10.9, 2.1)	50.0	2.70 (ddd, *J* = 10.7, 6.8, 3.6)	48.3
5	3.32 (m)	32.0	2.99 (m)	37.0
6α	1.45 (td, *J* = 10.8, 3.4)	42.0	1.65 (td, *J* = 12.6, 4.4)	39.1
6β	2.33 (m)		1.95 (dd, *J* = 12.6, 6.6)	
7	4.14 (q, *J* = 3.4)	75.0	4.24 (q, *J* = 4.4)	75.1
8	2.30 (m)	43.6	2.05 (m)	42.4
9	2.88 (t, *J* = 10.9)	46.2	2.22 (m)	44.9
10	1.28 (d, *J* = 7.0)	14.4	1.13 (d, *J* = 7.2)	14.2
11		170.6		175.4
11-O*Me*	3.78 (s)	52.7	3.67 (s)	52.2
1′				130.4
2′			8.00 (d, *J* = 7.5)	129.7
3′			7.45 (t, *J* = 7.5)	128.7
4′			7.57 (t, *J* = 7.5)	133.3
5′			7.45 (t, *J* = 7.5)	128.7
6′			8.00 (d, *J* = 7.5)	129.7
7′				166.8

^a^ The chemical shifts are expressed in δ ppm. The coupling constants (*J*) are expressed in Hz.

**Table 2:** ^1^H-NMR and ^1^^3^C-NMR spectroscopic data of compounds **3**–**4** (in CDCl_3_, 500 MHz) ^a^.

Compound	3		Compound	4	
Position	^1^H ^a^	^13^C	Position	^1^H^a^	^13^C
1		174.1	1		173.5
2			2		
3	5.63 (d, *J* = 2.6)	98.3	3	5.59 (d, *J* = 2.5)	98.1
4	2.60 (dd, *J* = 11.6, 2.6)	50.1	4	2.59 (dd, *J* = 11.6, 2.5)	50.0
5	3.31 (m)	32.0	5	3.33 (m)	32.1
6α	1.42 (m)	42.3	6α	1.42 (m)	42.3
6β	2.34 (dd, *J* = 13.6, 7.6)		6β	2.35 (dd, *J* = 13.8, 7.6)	
7	4.12 (m)	75.1	7	4.13 (m)	75.1
8	2.27(m)	43.7	8	2.29 (m)	43.6
9	2.79 (dd, *J* = 11.6, 10.1)	46.2	9	2.77 (dd, *J* = 11.6, 10.1)	46.1
10	1.29 (d, *J* = 6.9)	14.6	10	1.29 (d, *J* = 6.8)	14.6
11		169.7	11		169.8
11-O*Me*	3.82 (s)	52.7	11-O*Me*	3.77, s	52.6
1′	4.93 (d, *J* = 5.6)	95.7	1′		173.3
2′			2′		
3′	7.40 (d, *J* = 1.2)	151.5	3′α	4.45 (dd, *J* = 11.3, 3.3)	67.5
			3′β	4.25 (dd, *J* = 11.3, 9.8)	
4′		111.8	4′	2.52 (td, *J* = 9.8, 3.3)	46.1
5′	2.93 (q, *J* = 8.5)	31.7	5′	2.84 (m)	37.4
6′α	1.37 (m)	38.2	6′α	1.37 (m)	37.3
6′β	2.42 (dd, *J* = 14.3, 7.4)		6′β	2.30 (m)	
7′	4.20 (m)	80.4	7′	4.23 (m)	81.0
8′	1.97 (m)	40.9	8′	2.40 (m)	43.9
9′	1.89 (td, *J* = 8.5, 5.6)	46.8	9′	2.64 (dd, *J* = 11.2, 9.6)	46.2
10′	1.08 (d, *J* = 6.9)	13.9	10′	1.19 (d, *J* = 6.8)	14.7
11′		167.6	11′		171.2
11′-O*Me*	3.71 (s)	51.4	11′-O*Me*	3.74 (s)	52.6

^a^ The chemical shifts are expressed in δ ppm. The coupling constants (*J*) are expressed in Hz.

**Table 3:** *In vitro* inhibition of nitric oxide production of compounds **1**–**20** on NO production by LPS stimulated in RAW264.7 cells.

Compounds	CC_50_ (μg/mL) ^a^	IC_50_ (μg/mL) ^a^	Compounds	CC_50_ (μg/mL) ^a^	IC_50_ (μg/mL) ^a^
**1**	>100 ^b^	>100	**11**	>100 ^b^	>100
**2**	>100 ^b^	>100	**12**	>100 ^b^	76.18 ± 2.42 ***
**3**	>100 ^b^	>100	**13**	>100 ^b^	>100
**4**	>100 ^b^	>100	**14**	>100 ^b^	72.91 ± 4.97 **
**5**	>100 ^b^	>100	**15**	>100 ^b^	95.16 ± 1.20 ***
**6**	>100 ^b^	>100	**16**	>100 ^b^	>100
**7**	>100 ^b^	86.27 ± 3.45 ***	**17**	>100 ^b^	>100
**8**	>100 ^b^	>100	**18**	>100 ^b^	>100
**9**	>100 ^b^	>100	**19**	>100 ^b^	>100
**10**	>100 ^b^	9.18 ± 1.90 ***	**20**	>100 ^b^	>100
**11**	>100 ^b^	>100	Indomethacin	>100 ^b^	46.71 ± 3.14

^a^ The values shown are the mean ± SD of data from three independent experiments; ^b^ CC_50_ could not be obtained because of a cell survival rate > 88% at 100-6.25 μg/mL; ** *p* < 0.01, *** *p* < 0.001 vs Indomethacin.

## Supplementary Materials

The following are available online: 1D- and 2D-NMR, as well as HR-ESI-MS spectra of Compounds **1**–**4**.
