# Supplementary material for: Four New Iridoid Metabolites Have Been Isolated from the Stems of Neonauclea reticulata (Havil.) Merr. with Anti-Inflammatory Activities on LPS-Induced RAW264.7 Cells
Source: Molecules. 2019 Nov 23;24(23):4271. doi: 10.3390/molecules24234271 (PMC6930649; doi:10.3390/molecules24234271)
Supplement: Supplementary file 1 [file molecules-24-04271-s001.pdf]

Supplementary Materials

Four New Iridoid Metabolites Have Been Isolated from the Stems of *Neonauclea reticulata* (Havil.) Merr. with Anti-Inflammatory Activities on LPS-Induced RAW264.7 Cells

Fang-Pin Chang [1], Shyh-Shyun Huang [2], Tzong-Huei Lee [3], Chi-I Chang [4], Tzong-Fu Kuo [5] Guan-Jhong Huang [6,*] and Yueh-Hsiung Kuo [1,6,7,8,*]

[1] The Ph.D Program for Cancer Biology and Drug Discovery, China Medical University and Academia Sinica, Taichung 404, Taiwan; u101049002@cmu.edu.tw
[2] School of Pharmacy, China Medical University, Taichung 404, Taiwan; sshuang@mail.cmu.edu.tw
[3] Institute of Fisheries Science, National Taiwan University, Taipei 106, Taiwan; thlee1@ntu.edu.tw
[4] Department of Biological Science and Technology, National Pingtung University of Science and Technology,Pingtung 912, Taiwan; changchii@mail.npust.edu.tw
[5] Department of Post-Baccalaureate Veterinary Medicine, Asia University, Taichung 413, Taiwan; tzongfu@asia.edu.tw
[6] Department of Chinese Pharmaceutical Sciences and Chinese Medicine Resources, China Medical University, Taichung 404, Taiwan.; gjhuang@mail.cmu.edu.tw (G.-J.H.); kuoyh@mail.cmu.edu.tw (Y.-H.K.)
[7] Department of Biotechnology, Asia University, Taichung 413, Taiwan
[8] Chinese Medicine Research Center, China Medical University, Taichung 404, Taiwan
* Correspondence: gjhuang@mail.cmu.edu.tw (G.-J.H.); kuoyh@mail.cmu.edu.tw (Y.-H.K.); Tel.: +886-4-2205-3366 (ext. 5508) (G.-J.H.); +886-4-2205-3366 (ext. 5701) (Y.-H.K.)

† These authors contributed equally to this work

Table of contents

Figure S1. HR-ESI-MS spectrum of 1 ....S4

Figure S2. ^{1}H-NMR spectrum of 1 ($CDCl_3$, 500 MHz) S4

Figure S3. ^{13}C-NMR spectrum of 1 ($CDCl_3$, 125 MHz) S5

Figure S4. DEPT spectrum of 1 ($CDCl_3$, 125 MHz) S5

Figure S5. HSQC spectrum of 1 ($CDCl_3$, 500 MHz) S6

Figure S6. COSY spectrum of 1 ($CDCl_3$, 500 MHz) S6

Figure S7. HMBC spectrum of 1 ($CDCl_3$, 500 MHz) S7

Figure S8. NOESY spectrum of 1 ($CDCl_3$, 500 MHz) S7

Figure S9. HR-ESI-MS spectrum of 2 ....S8

Figure S10. ^{1}H-NMR spectrum of 2 ($CDCl_3$, 500 MHz) S8

Figure S11. ^{13}C-NMR spectrum of 2 ($CDCl_3$, 125 MHz) S9

Figure S12. HSQC spectrum of 2 ($CDCl_3$, 500 MHz) S9

Figure S13. COSY spectrum of 2 ($CDCl_3$, 500 MHz) S10

Figure S14. HMBC spectrum of 2 ($CDCl_3$, 500 MHz) S10

Figure S15. NOESY spectrum of 2 ($CDCl_3$, 500 MHz) S11

Figure S16. HR-ESI-MS spectrum of 3 ....S11

Figure S17. ^{1}H-NMR spectrum of 3 ($CDCl_3$, 500 MHz) S12

Figure S18. ^{13}C-NMR spectrum of 3 ($CDCl_3$, 125 MHz) S12

Figure S19. DEPT spectrum of 3 ($CDCl_3$, 125 MHz) S13

Figure S20. HSQC spectrum of 3 ($CDCl_3$, 500 MHz) S13

Figure S21. COSY spectrum of 3 ($CDCl_3$, 500 MHz) S14

Figure S22. HMBC spectrum of 3 ($CDCl_3$, 500 MHz) S14

Figure S23. NOESY spectrum of 3 ($CDCl_3$, 500 MHz) S15

Figure S25. ^{1}H-NMR spectrum of 4 ($CDCl_3$, 500 MHz) S16

Figure S26. ^{13}C-NMR spectrum of 4 ($CDCl_3$, 125 MHz) S16

Figure S27. DEPT spectrum of 4 ($CDCl_3$, 125 MHz) S17

Figure S28. HSQC spectrum of 4 ($CDCl_3$, 500 MHz) S17

Figure S29. COSY spectrum of 4 ($CDCl_3$, 500 MHz) S18

Figure S30. HMBC spectrum of 4 ($CDCl_3$, 500 MHz) S18

Figure S31. NOESY spectrum of 4 ($CDCl_3$, 500 MHz) S19

Figure S32. The *in vitro* cell viability (%) & nitric oxide production (%) of compound (7), (10), (12), (14), (15) & indomethacin for 24 h.. S20

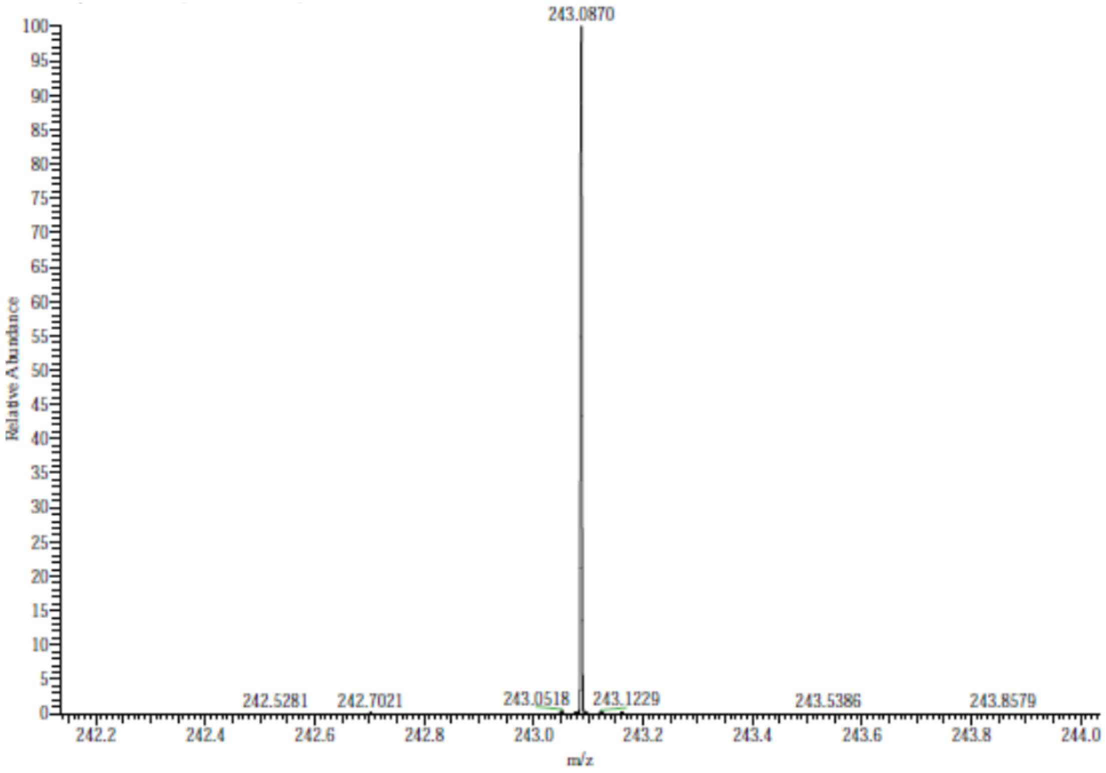

Figure S1. HR-ESI-MS spectrum of **1**

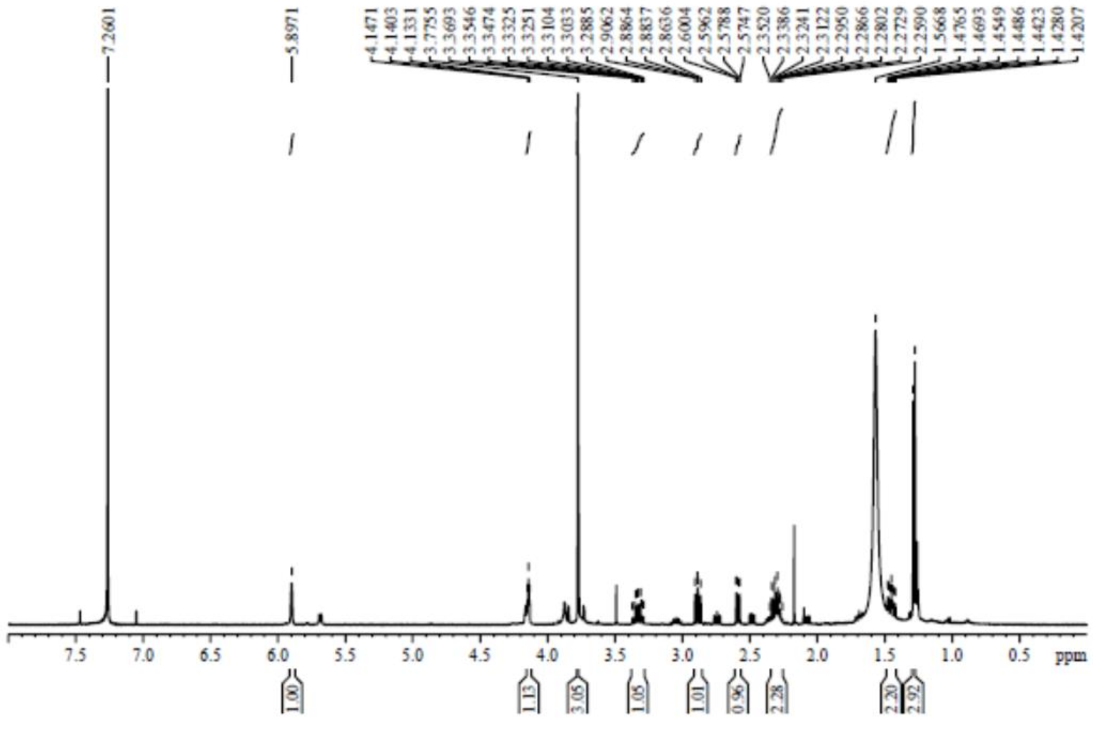

Figure S2. ^{1}H-NMR spectrum of **1** ($CDCl_3$, 500 MHz)

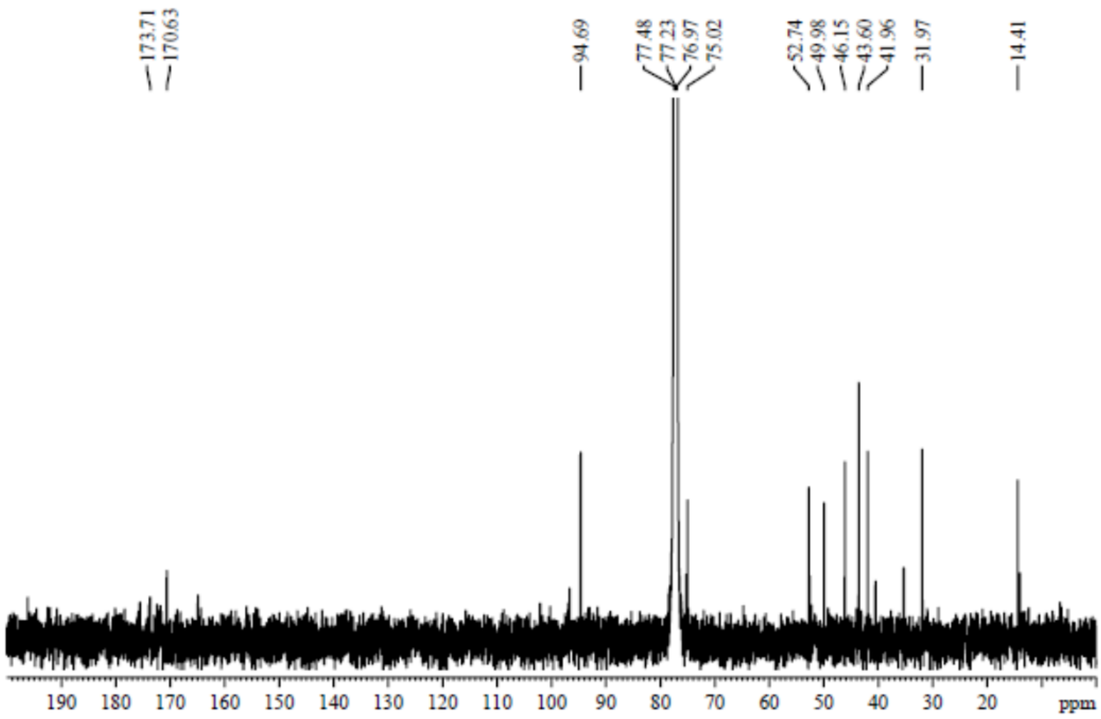

Figure S3. ^{13}C-NMR spectrum of **1** ($CDCl_3$, 125 MHz)

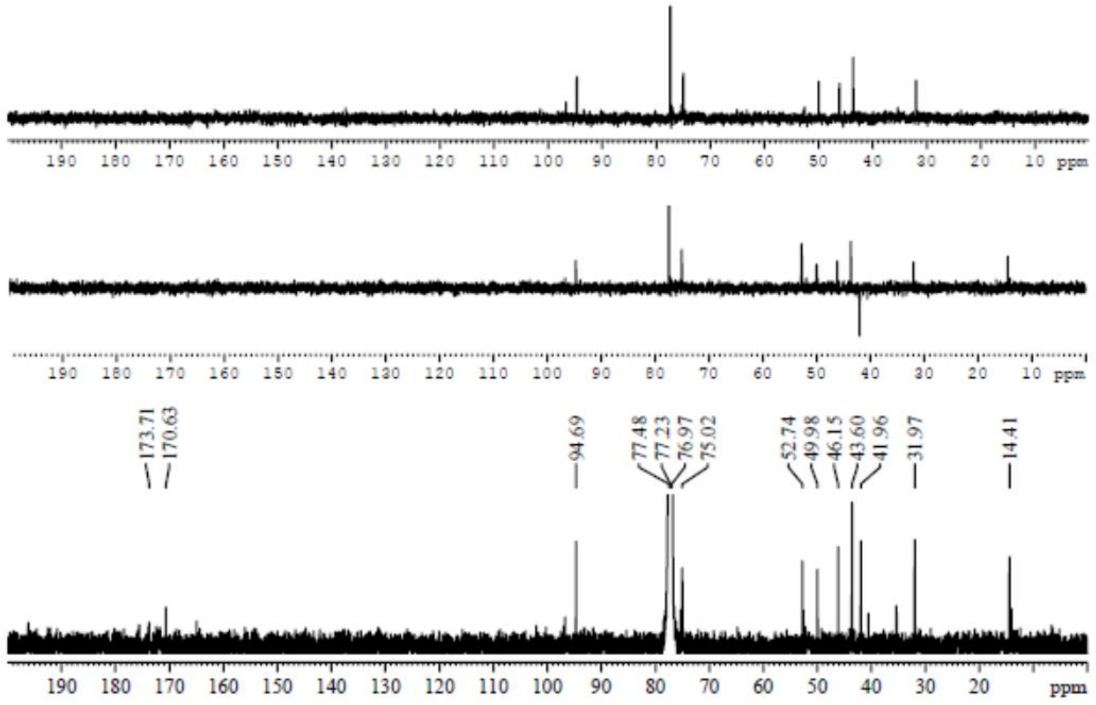

Figure S4. DEPT spectrum of **1** ($CDCl_3$, 125 MHz)

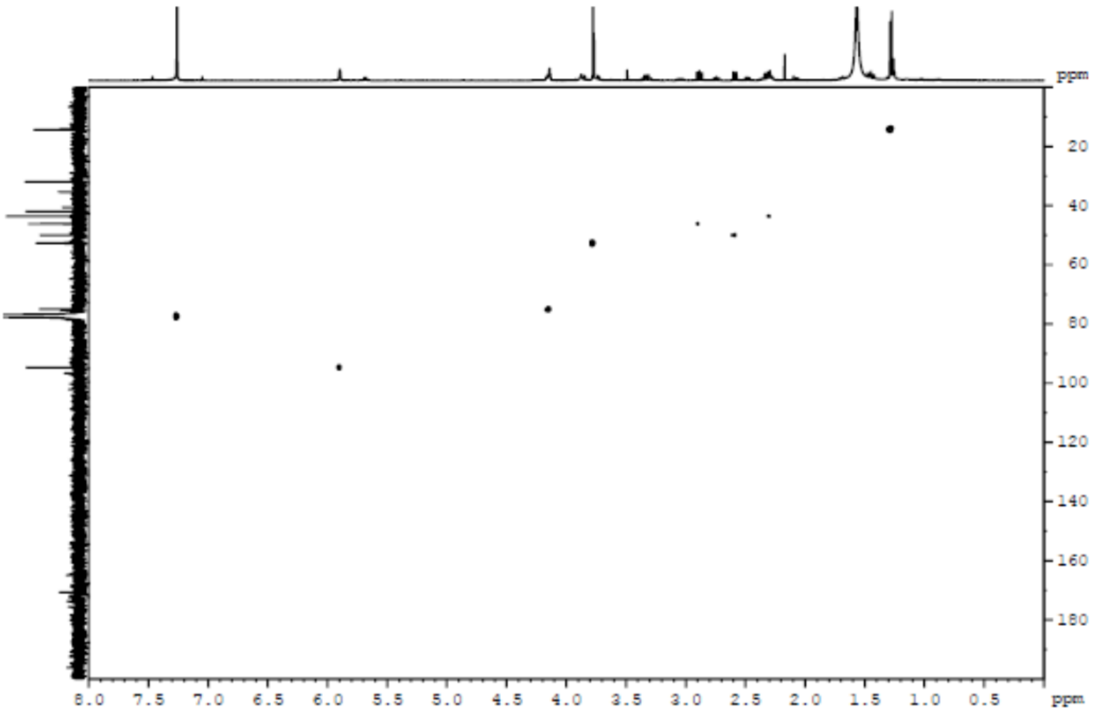

Figure S5. HSQC spectrum of **1** ($CDCl_3$, 500 MHz)

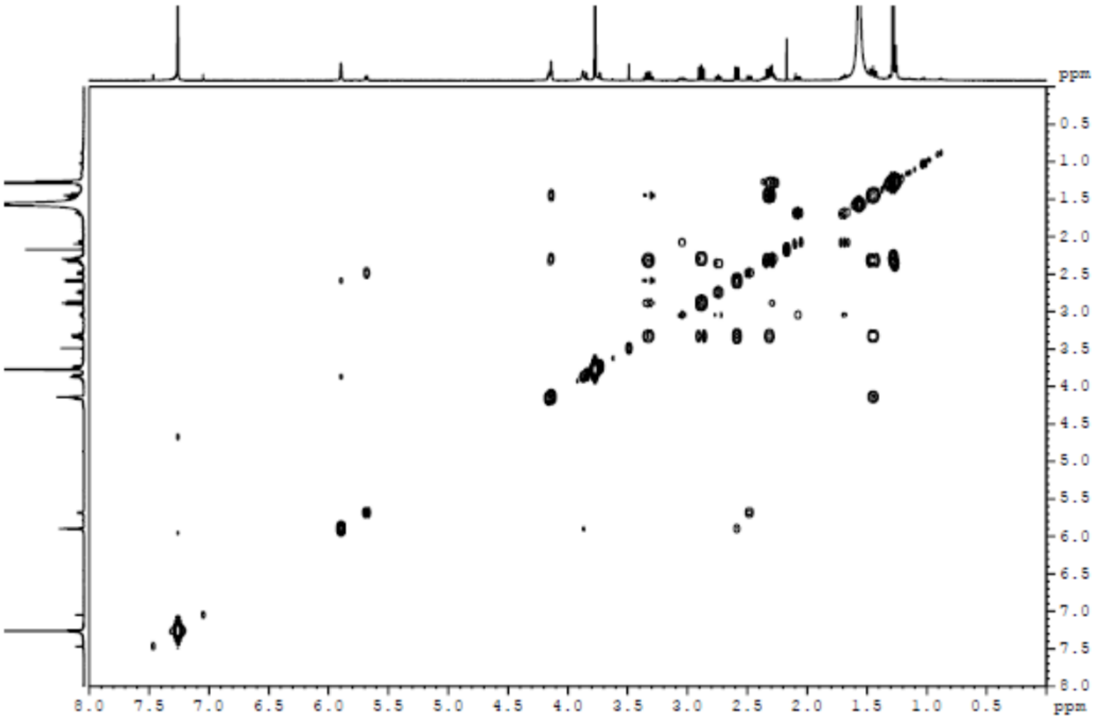

Figure S6. COSY spectrum of **1** ($CDCl_3$, 500 MHz)

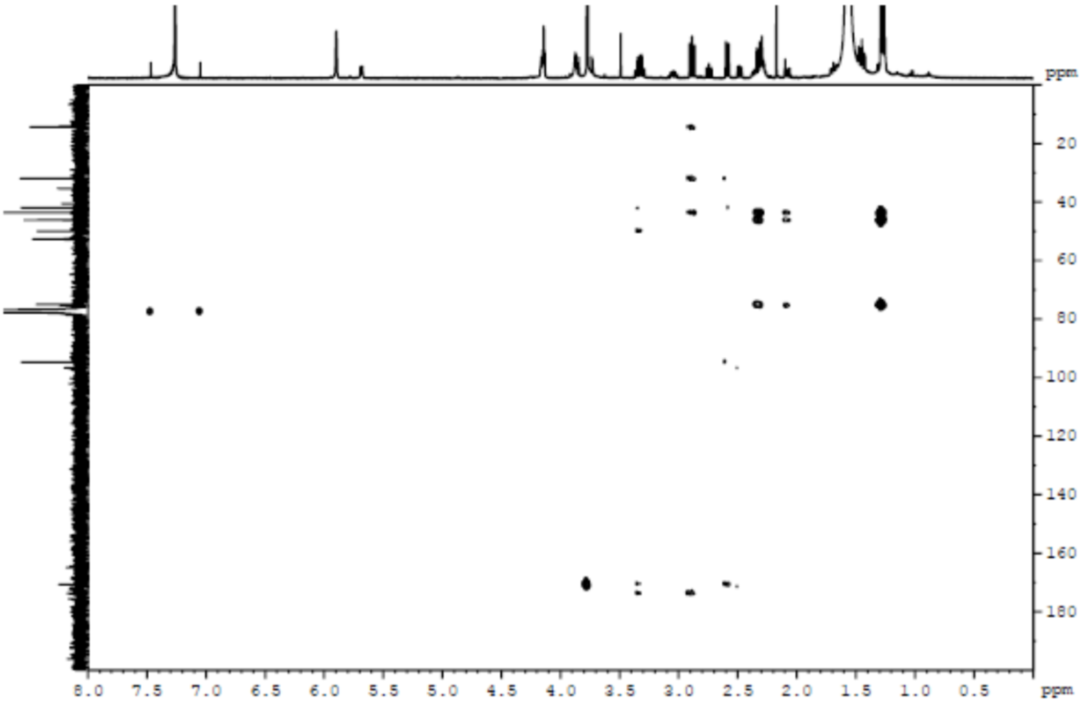

Figure S7. HMBC spectrum of **1** ($CDCl_3$, 500 MHz)

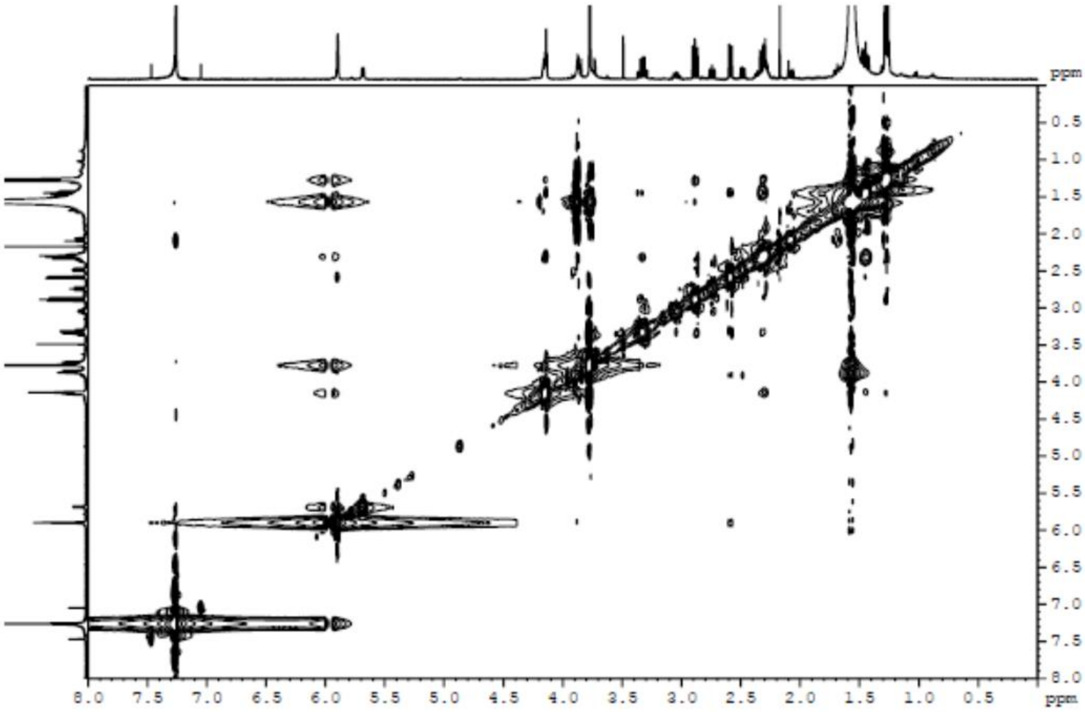

Figure S8. NOESY spectrum of **1** ($CDCl_3$, 500 MHz)

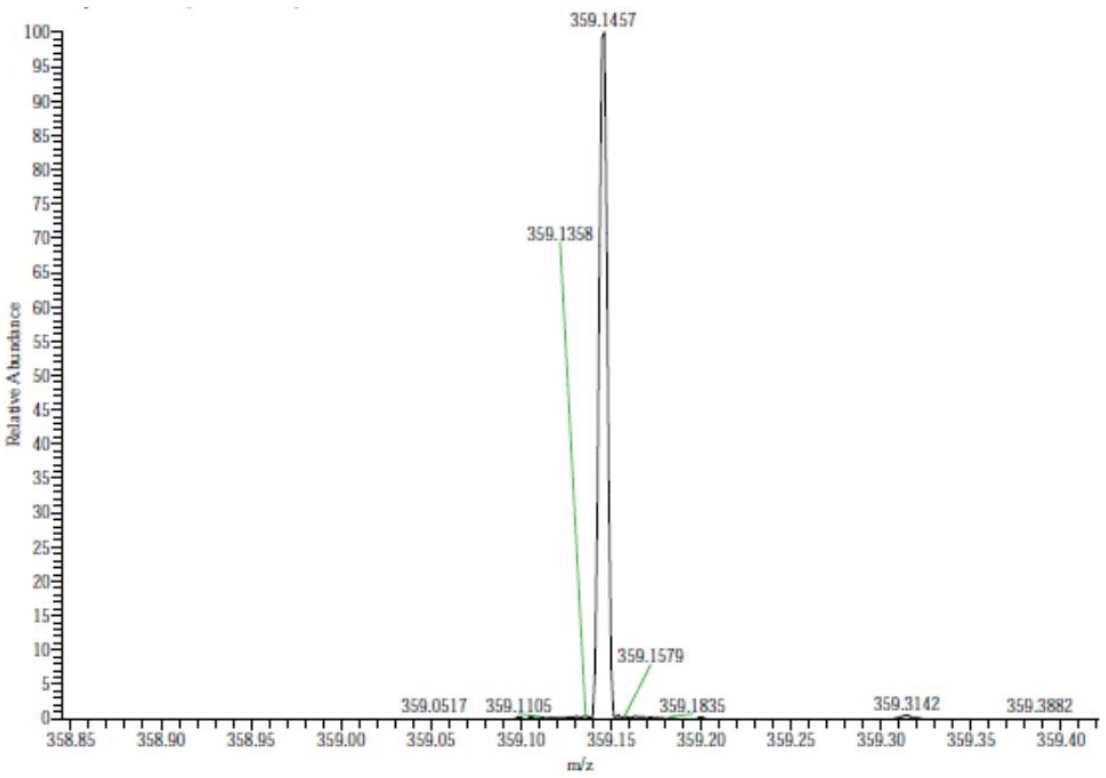

Figure S9. HR-ESI-MS spectrum of **2**

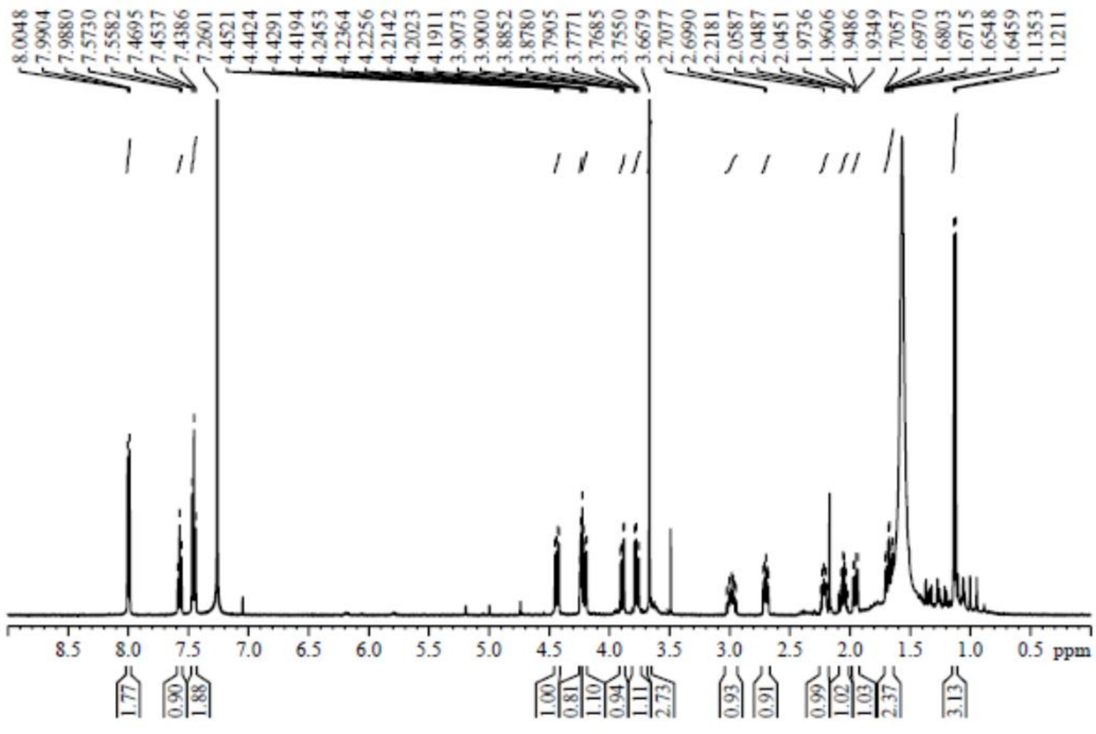

Figure S10. ^{1}H-NMR spectrum of **2** ($CDCl_3$, 500 MHz)

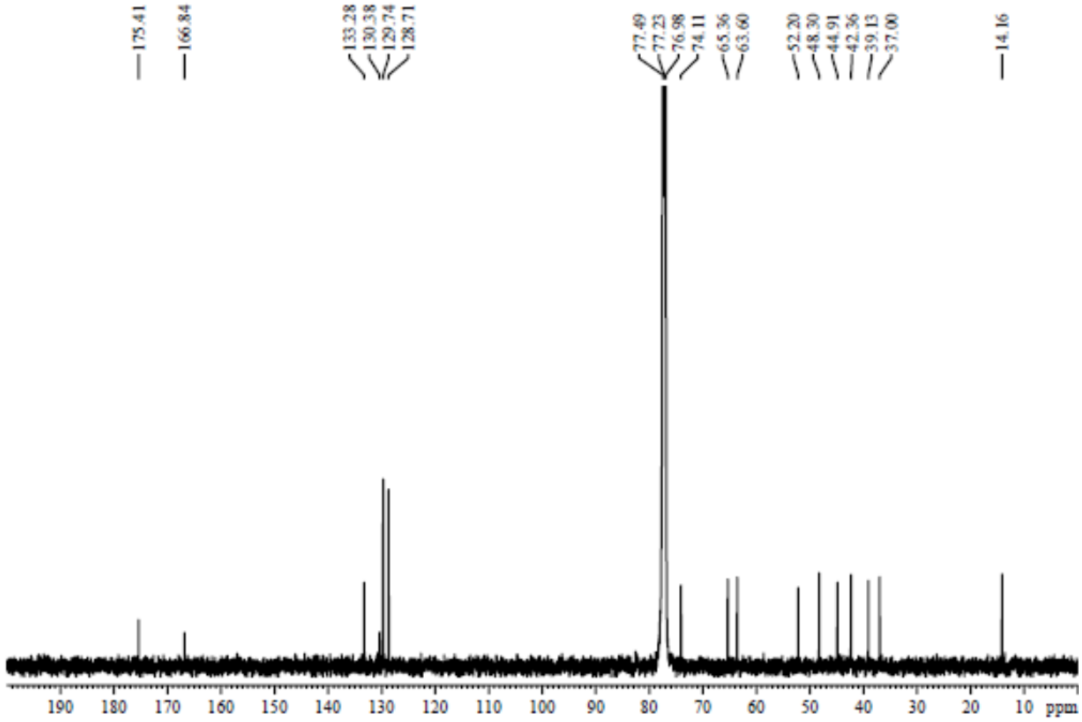

Figure S11. ^{13}C-NMR spectrum of **2** ($CDCl_3$, 125 MHz)

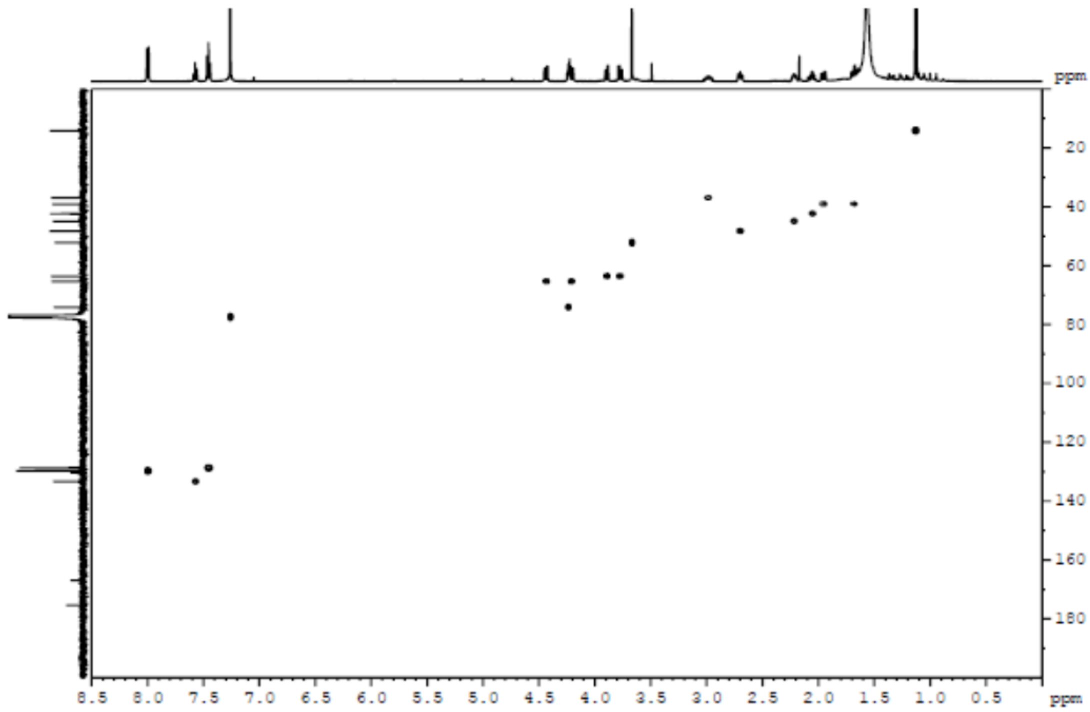

Figure S12. HSQC spectrum of **2** ($CDCl_3$, 500 MHz)

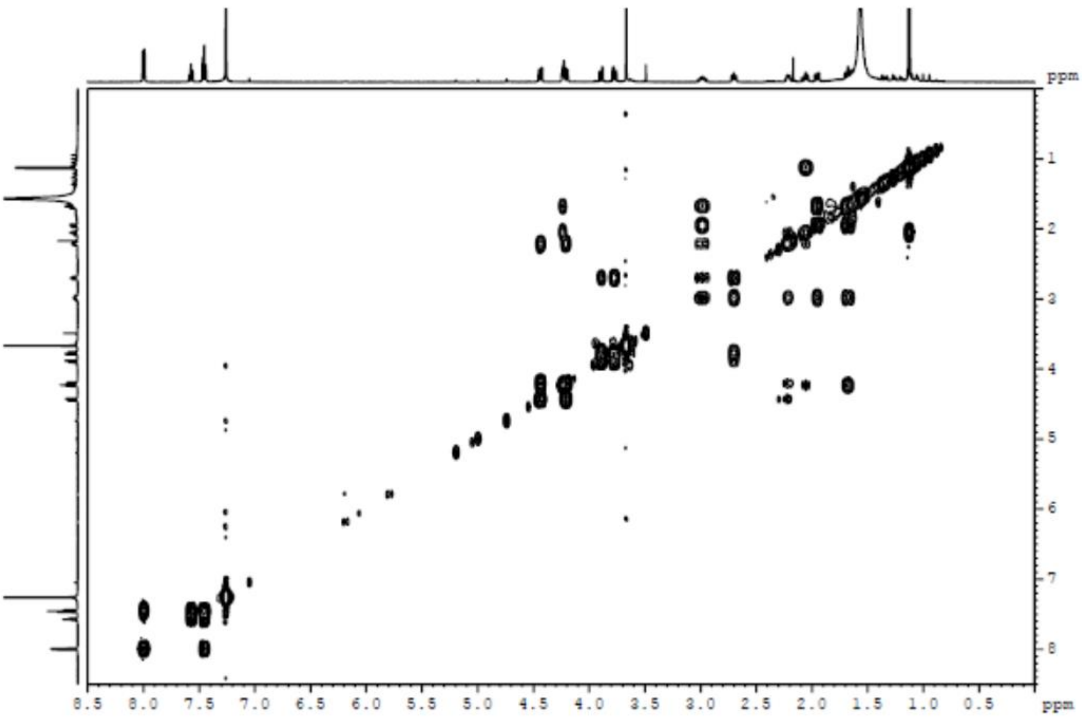

Figure S13. COSY spectrum of **2** ($CDCl_3$, 500 MHz)

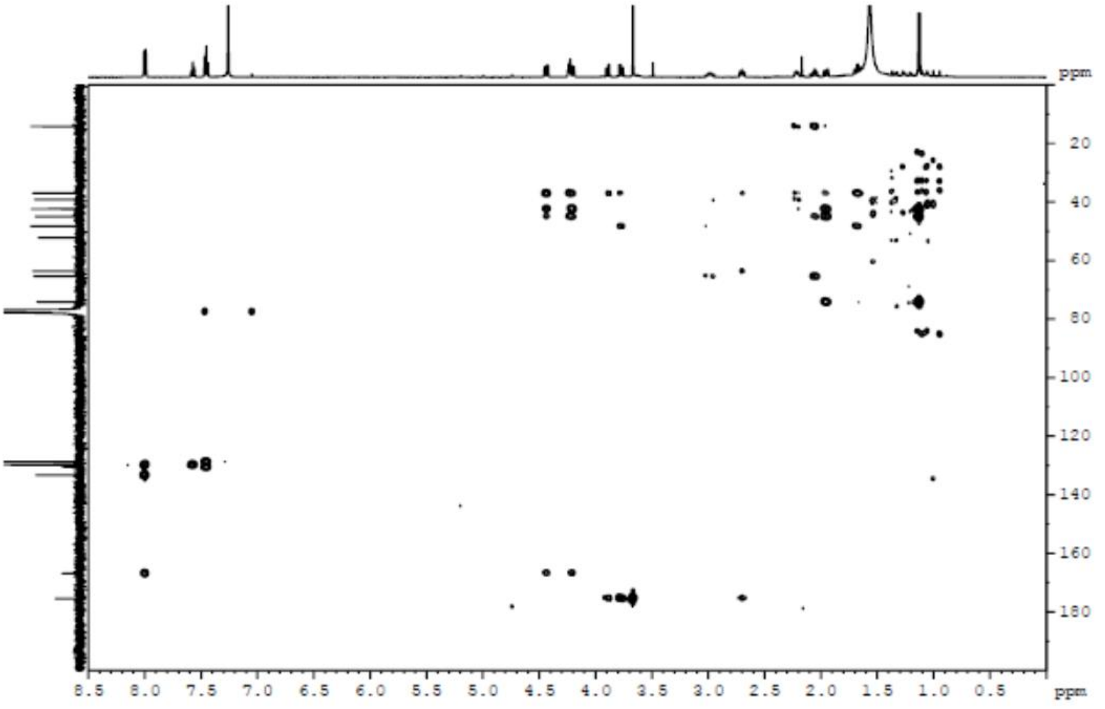

Figure S14. HMBC spectrum of **2** ($CDCl_3$, 500 MHz)

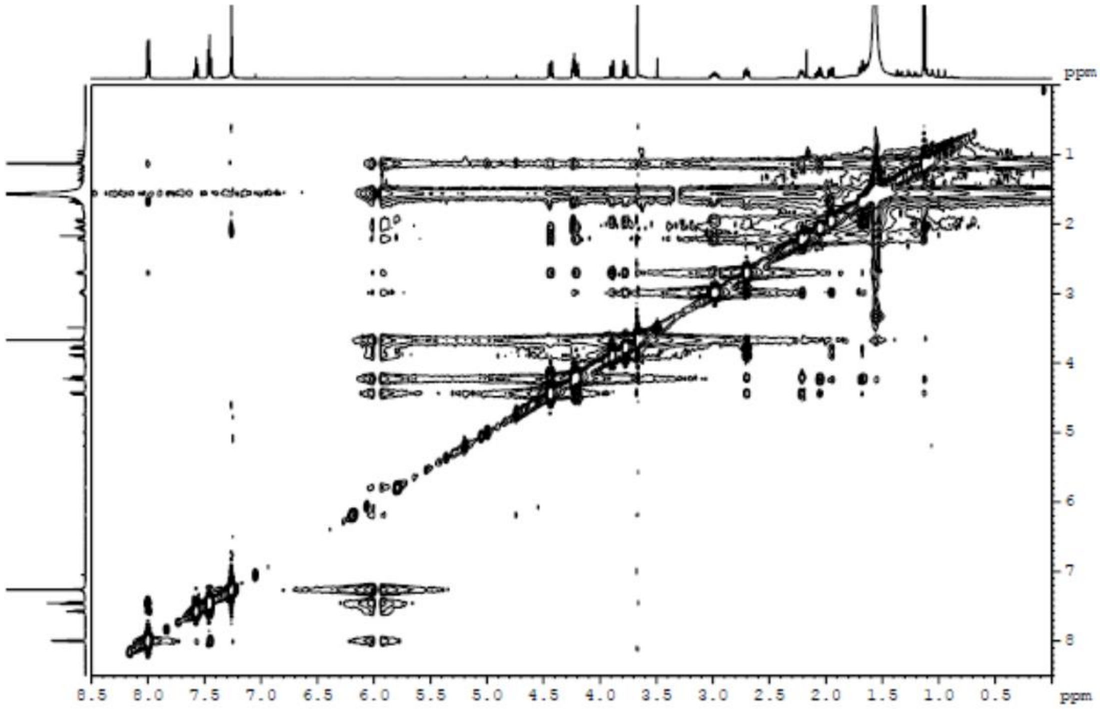

Figure S15. NOESY spectrum of **2** ($CDCl_3$, 500 MHz)

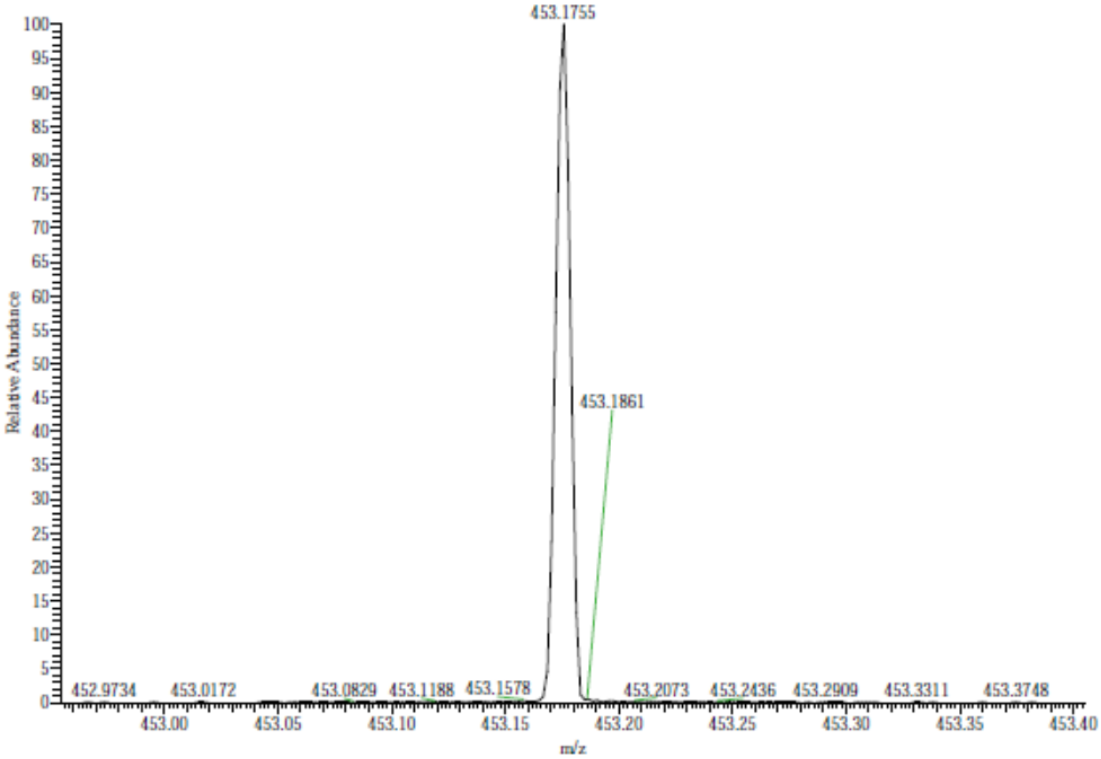

Figure S16. HR-ESI-MS spectrum of **3**

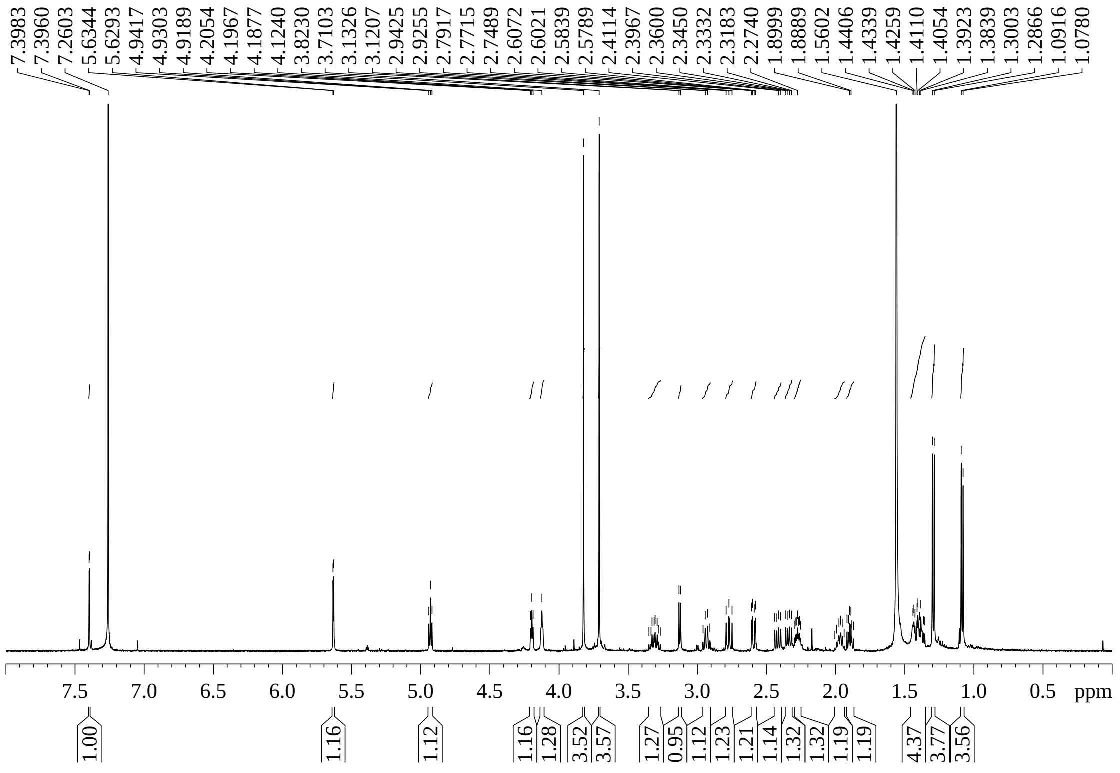

Figure S17. ^{1}H-NMR spectrum of **3** ($CDCl_3$, 500 MHz)

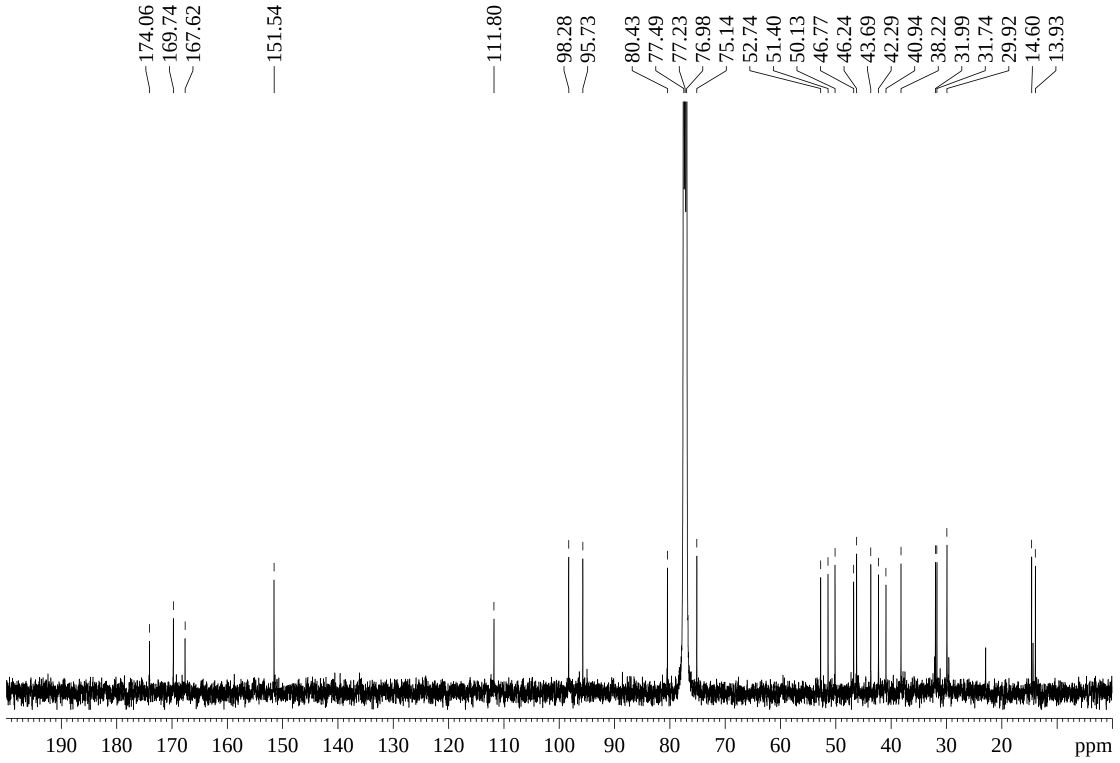

Figure S18. ^{13}C-NMR spectrum of **3** ($CDCl_3$, 125 MHz)

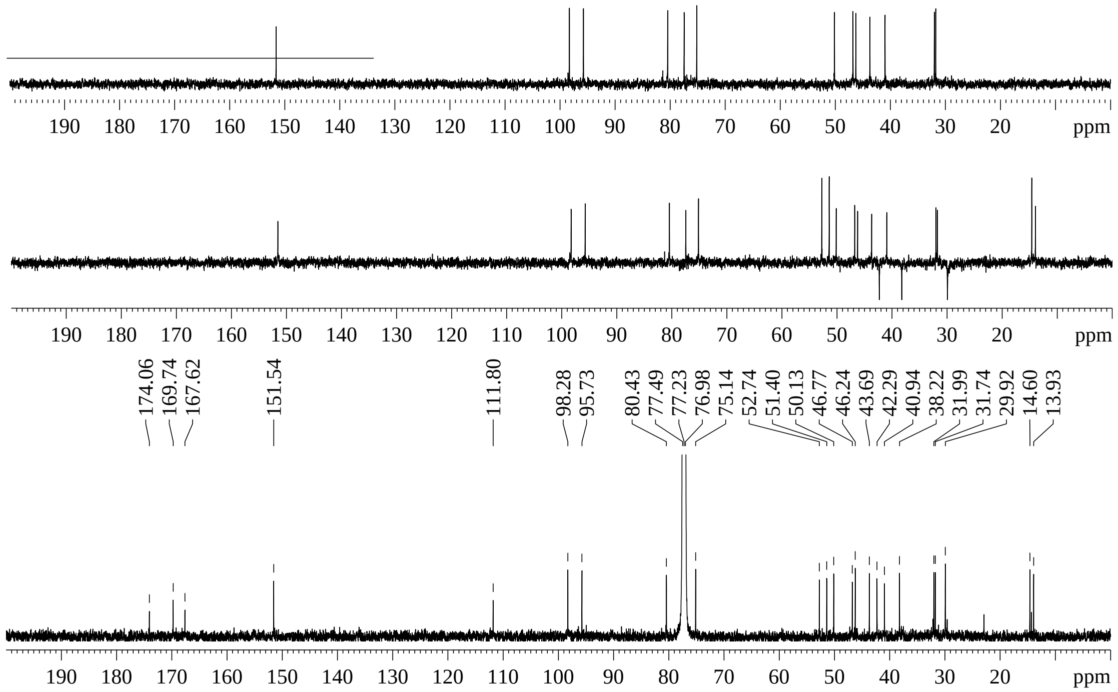

Figure S19. DEPT spectrum of **3** ($CDCl_3$, 125 MHz)

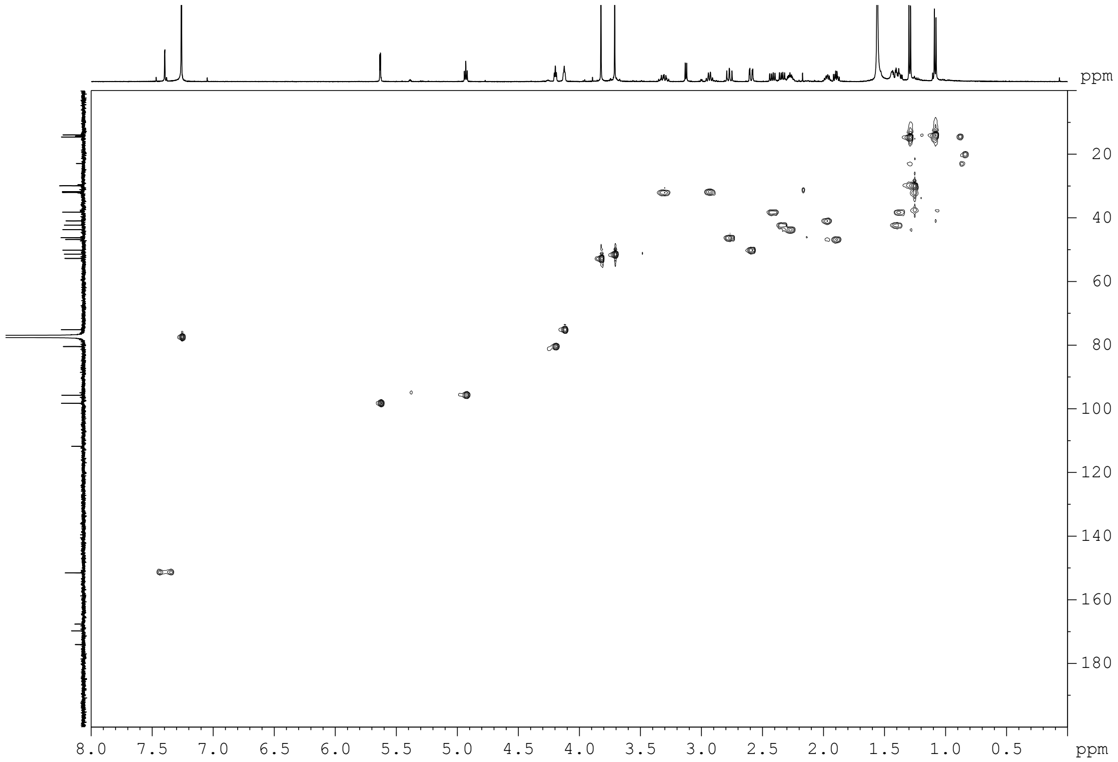

Figure S20. HSQC spectrum of **3** ($CDCl_3$, 500 MHz)

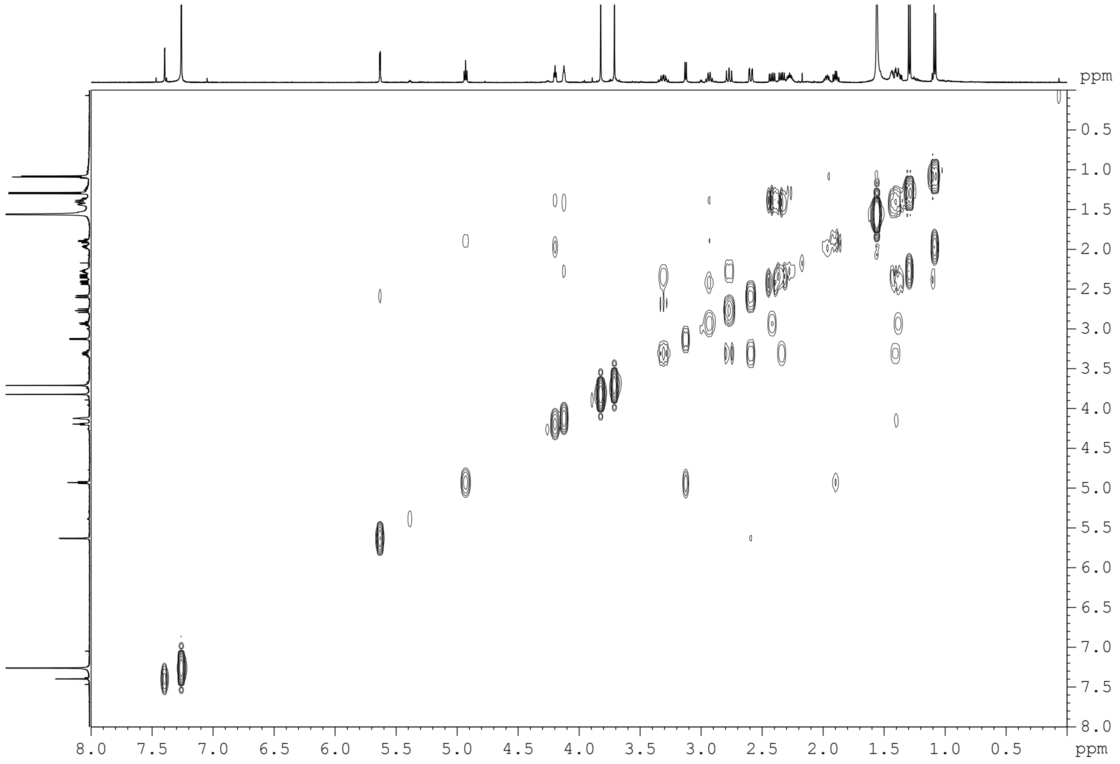

Figure S21. COSY spectrum of **3** ($CDCl_3$, 500 MHz)

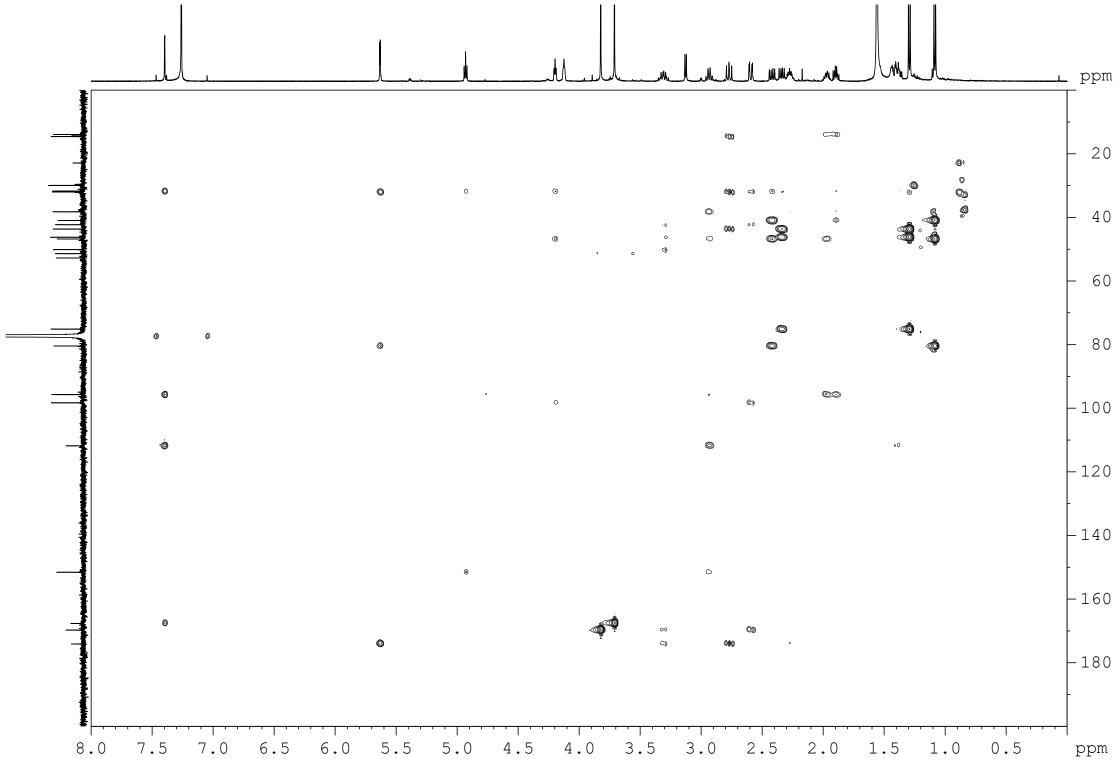

Figure S22. HMBC spectrum of **3** ($CDCl_3$, 500 MHz)

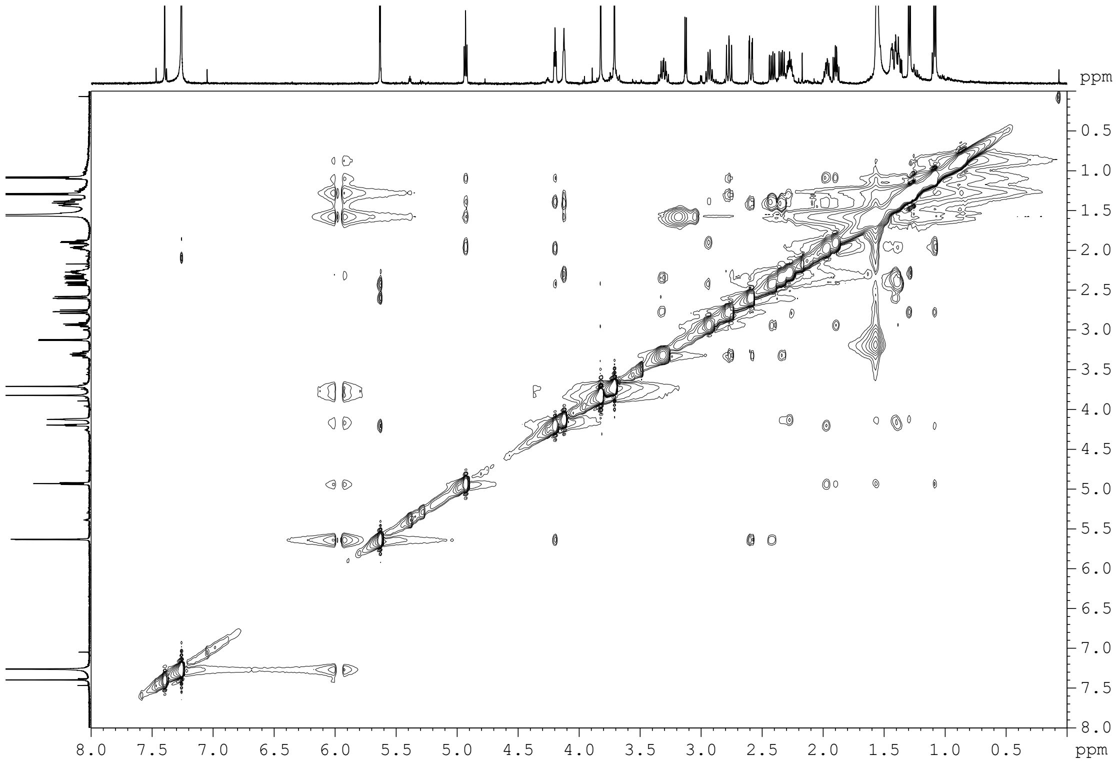

Figure S23. NOESY spectrum of **3** ($CDCl_3$, 500 MHz)

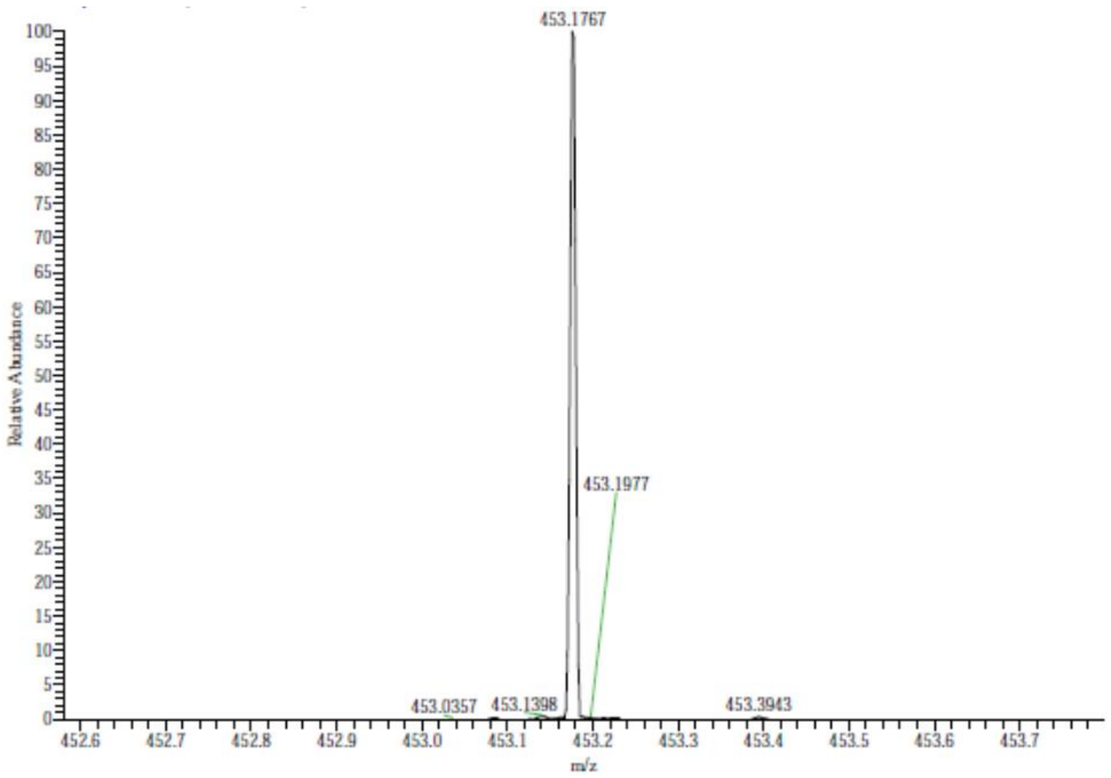

Figure S24. HR-ESI-MS spectrum of **4**

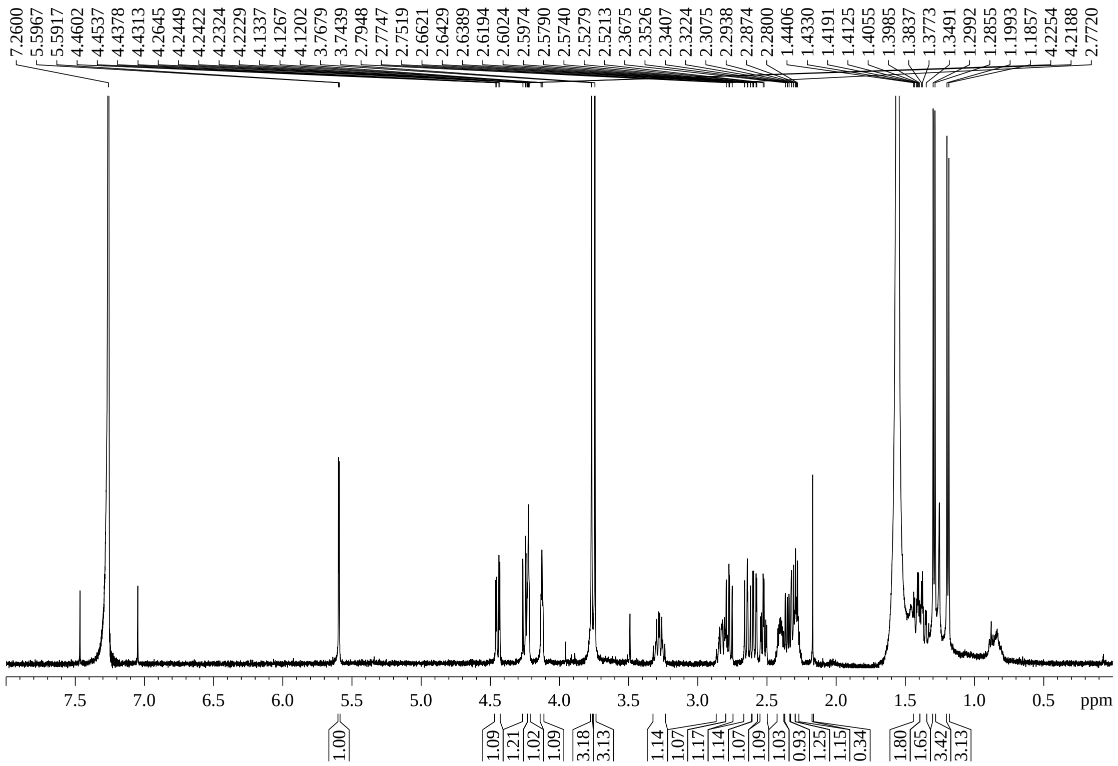

Figure S25. ^{1}H-NMR spectrum of **4** ($CDCl_3$, 500 MHz)

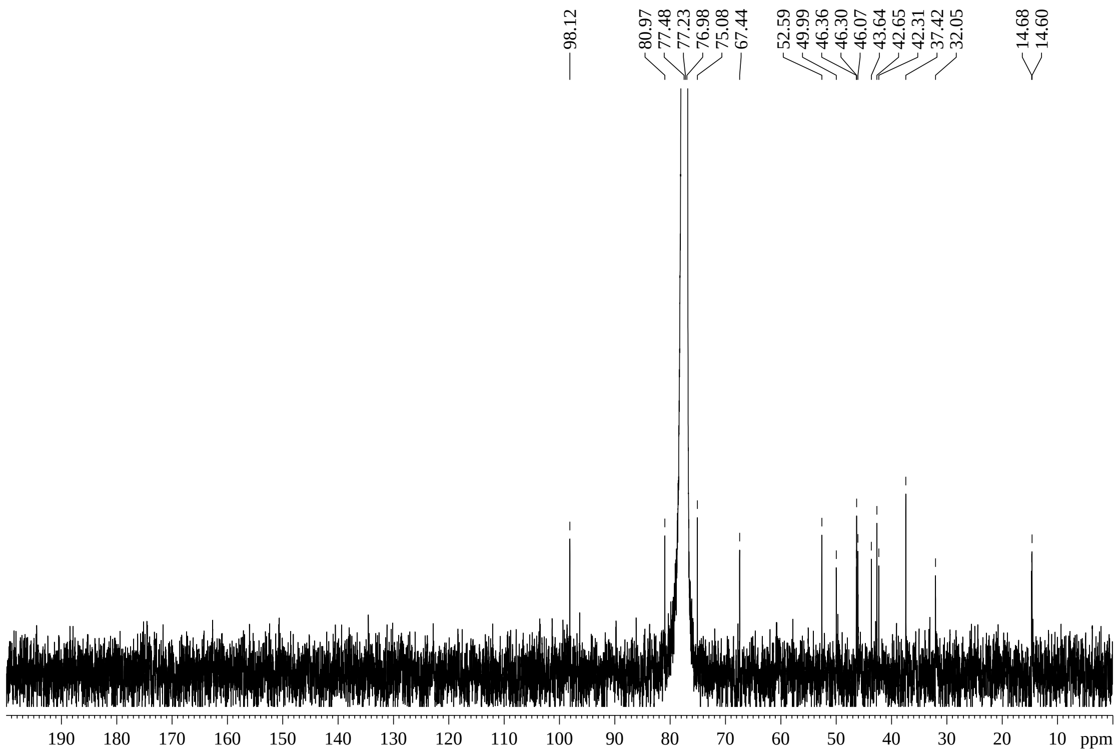

Figure S26. ^{13}C-NMR spectrum of **4** ($CDCl_3$, 125 MHz)

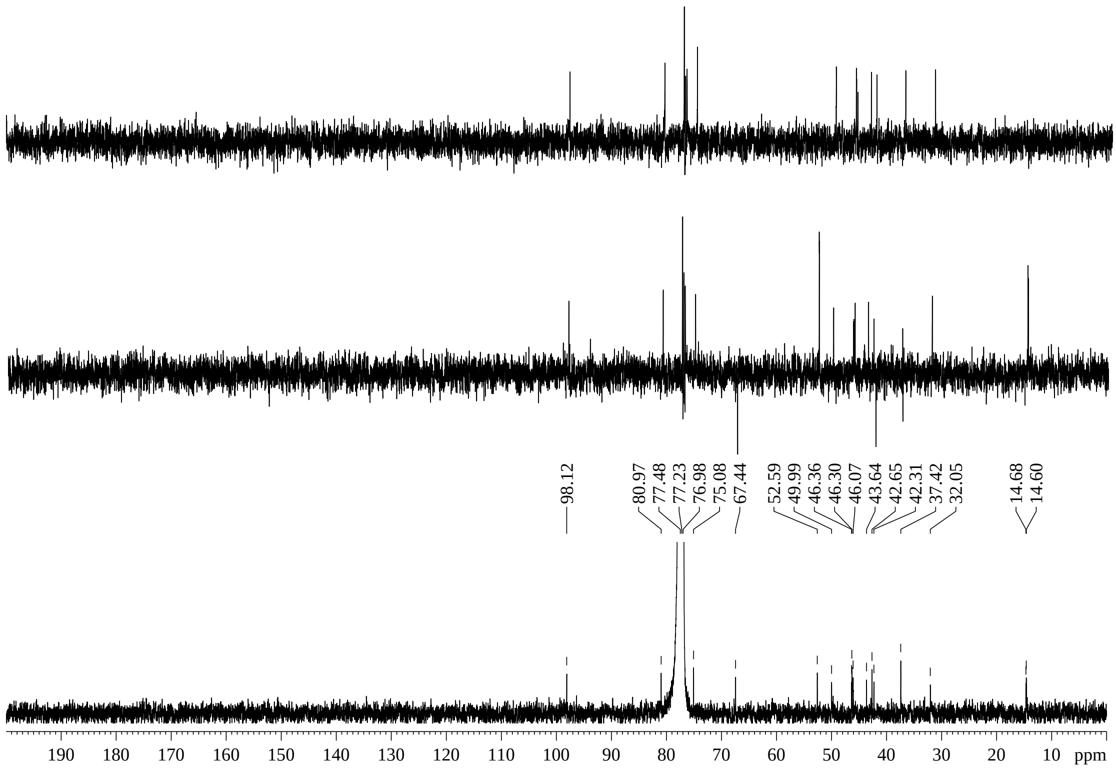

Figure S27. DEPT spectrum of **4** ($CDCl_3$, 125 MHz)

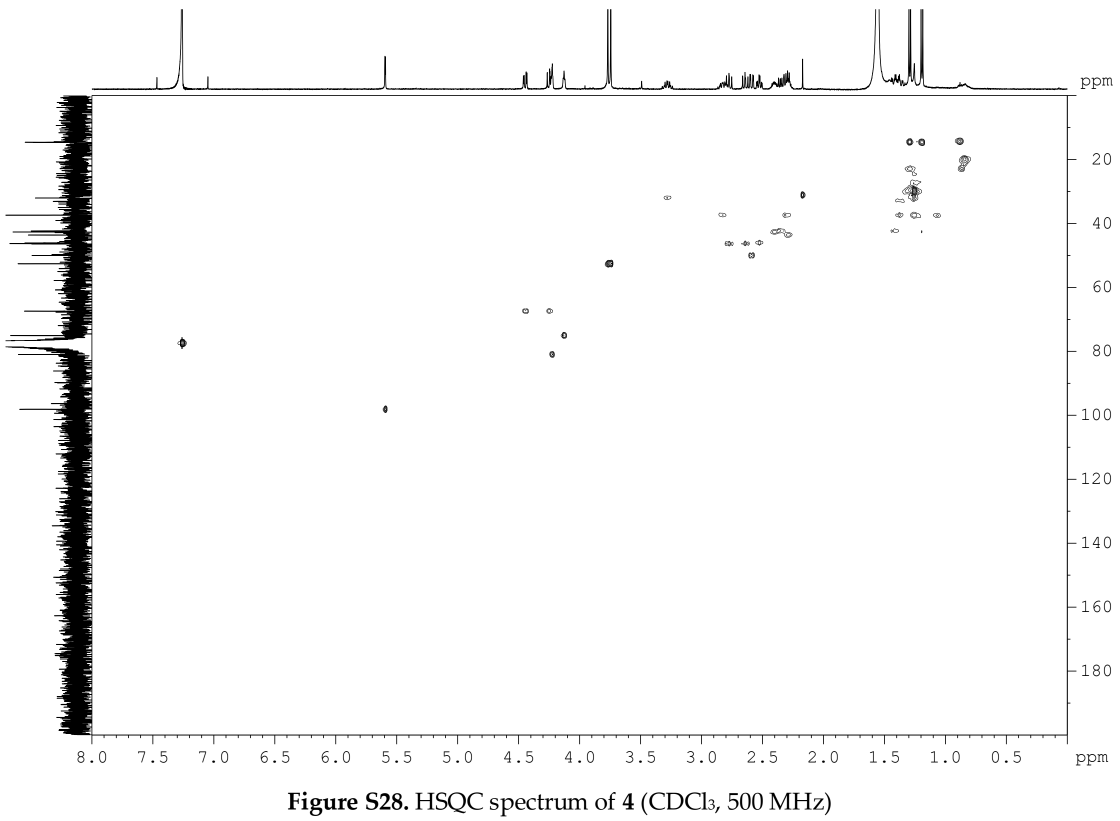

Figure S28. HSQC spectrum of **4** ($CDCl_3$, 500 MHz)

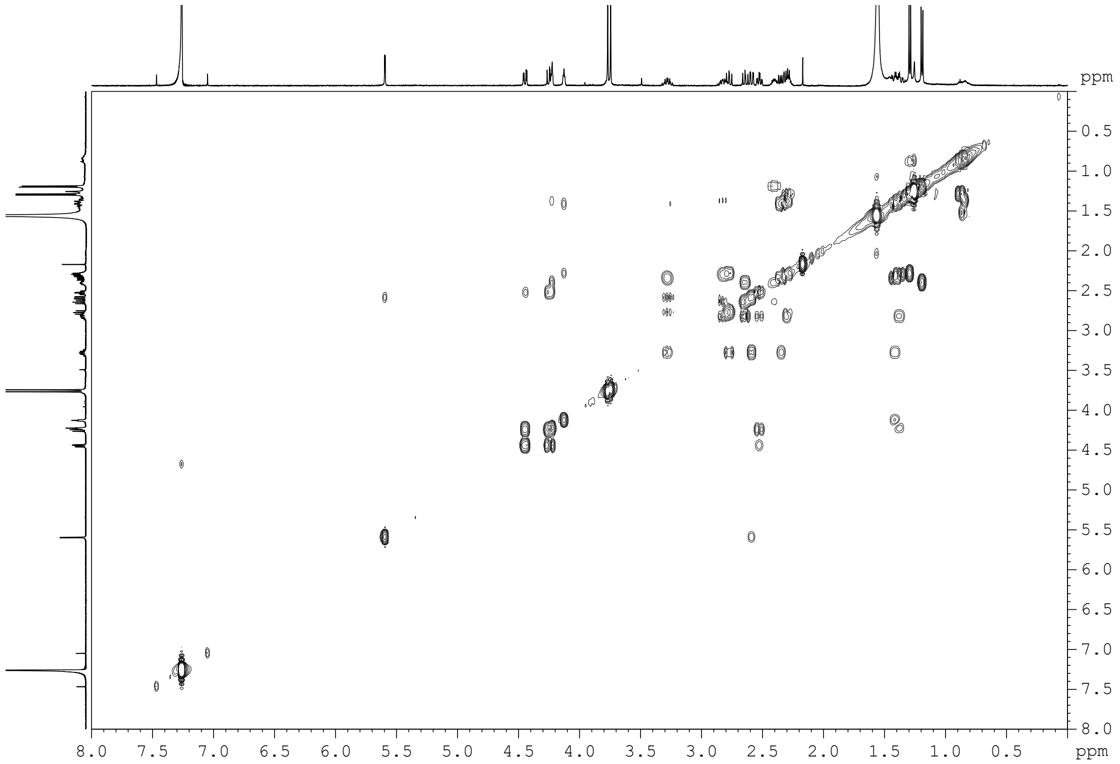

Figure S29. COSY spectrum of **4** ($CDCl_3$, 500 MHz)

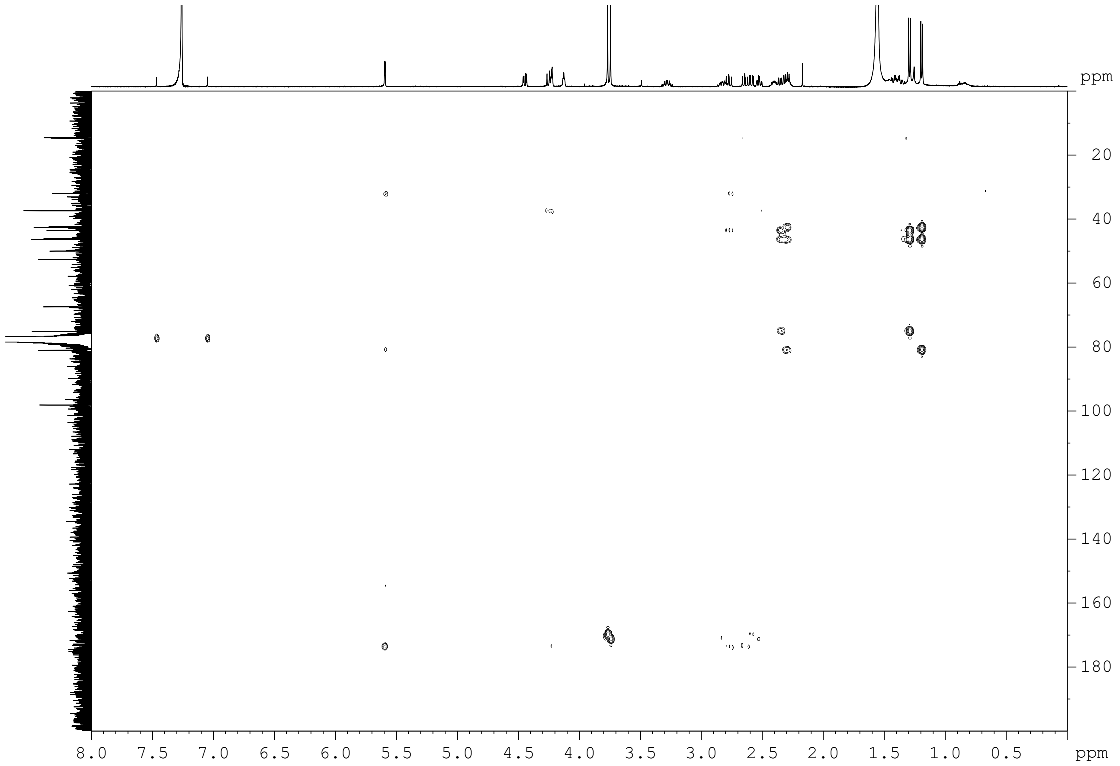

Figure S30. HMBC spectrum of **4** ($CDCl_3$, 500 MHz)

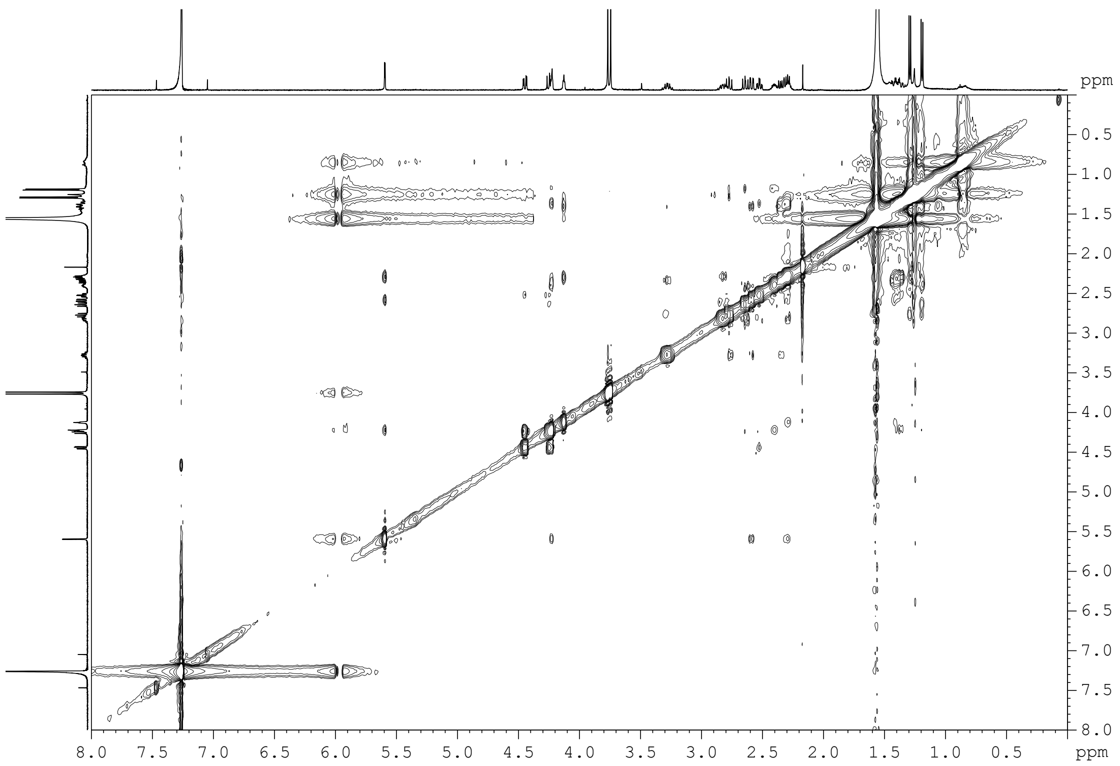

Figure S31. NOESY spectrum of **4** ($CDCl_3$, 500 MHz)

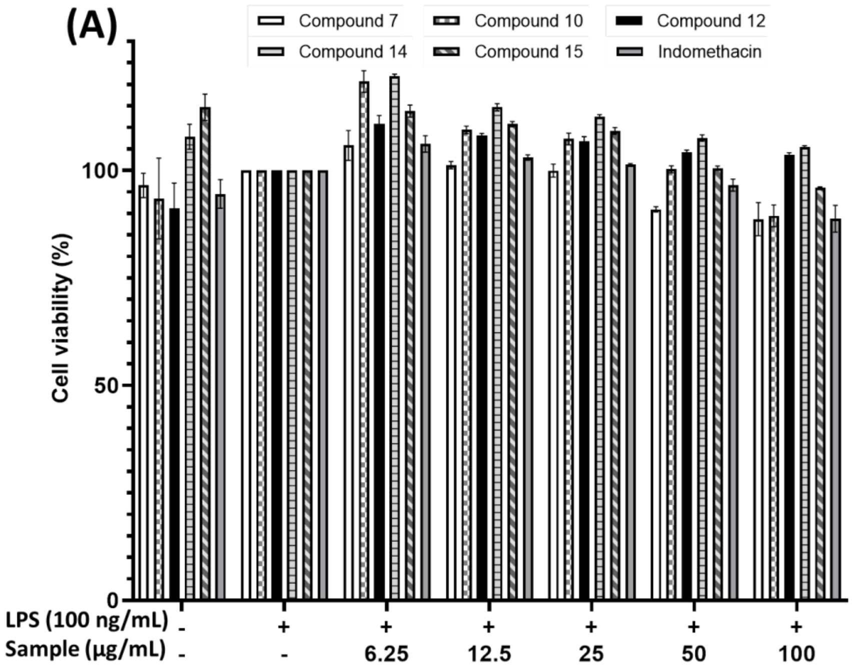

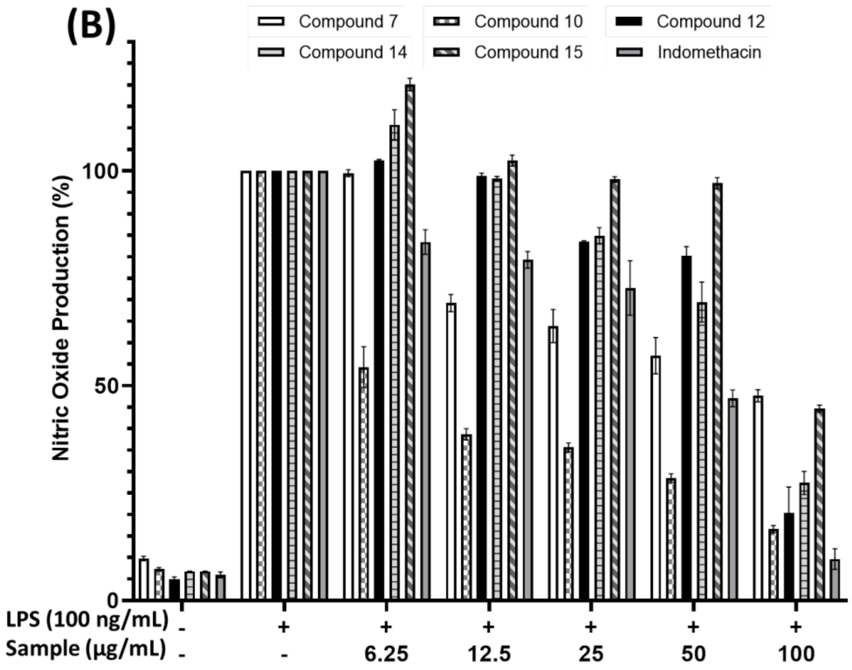

Figure S32. The *in vitro* cell viability (%) & nitric oxide production (%) of compound (**7**), (**10**), (**12**), (**14**), (**15**) & indomethacin for 24 h. (A) *in vitro* cell viability (%) was assessed in cells stimulated with LPS (100 ng/mL) was determined by MTT assay; (B) *in vitro* nitric oxide production (%) was determined using the Griess reagent method. The data represent the mean ± SD of triplicate experiments.
